# The viscosity solutions approach to swing options pricing under a regime-switching mean-reverting model

**DOI:** 10.1186/s13660-018-1908-3

**Published:** 2018-11-16

**Authors:** Lingjie Shao, Kaili Xiang, Yang Song

**Affiliations:** School of Economics and Mathematics, Southwestern University of Finance and Economics, Chengdu, China

**Keywords:** Swing options, Regime-switching, Mean-reverting, Variational inequalities, Viscosity solutions

## Abstract

In this paper, we study the valuation of swing options on electricity markets with local volume and refraction time constraints, under the setting that the dynamic of the underlying spot price is a 2-state regime-switching mean-reverting process. We derive the corresponding optimal multiple stopping problem, reduce it to a sequence of optimal single stopping problems, and further find that those value functions satisfy HJB variational inequalities subject to suitable conditions. Then after a prior estimation for the value functions, the viscosity solutions approach is adopted to get existence and uniqueness results in viscosity sense.

## Introduction

In real electricity markets, swing options provide the holder with rights to exercise repeatedly under certain constraints, such as limits on the local volume, the global volume, and refraction time etc. However, those trading rules add complexity to the associated valuation, which can be handled as stochastic optimal control problems. Besides the specification of option contracts, the other one of fundamental settings of the whole valuation procedure is the dynamic process characterizing the behavior of electricity prices, which remain high for a period of time after a jump, according to the empirical researches like [1–4] and [5]. Among various kinds of models, the regime-switching type is widely adopted. Wahab et al. [6] investigate the valuation problem under a three-state regime-switching model, where one regime is the Ornstein–Uhlenbeck type, and another two are Gaussian processes with constant coefficients. Wahab et al. [7] deal with a *n*-state regime-switching model where processes in all regime are geometric Brownian motion. Chiarella et al. [8] handle the valuation problem under a regime-switching forward curve model, and Bauerle et al. [9] choose a 2-state regime-switching model.

In all of [6–9], the lattice method is applied to price swing options, though with different choices of underlying price models. The lattice method concentrates on how to find the value function (a specific type of conditional expectation) by directly computing expectation iteratively with lattice. More specifically, in the light of stochastic control theory, the dynamic programming principle is satisfied by the value function leads us to the Bellman equation. Then lattices in different form can be applied to dealing with the conditional expectations arising in the corresponding Bellman’s equations. Different from the lattice method, another approach to tackling this problem is to seek the PDE characterization of the value function, which leads to the HJB variational inequalities. In this direction, Dahlgren et al. in [10] and [11] derive the variational inequalities and solve them by the finite difference method. In addition, basing on [12], Wilhelm and Winter [13] solve the related variational inequalities by finite element method. Besides, according to [14] and [15], the viscosity solutions approach is a powerful tool to deal with stochastic optimal control problems, and it is well known that the value function is the unique viscosity solution to the HJB-type equation or variational inequality subject to certain conditions, and that this approach can also help studying properties (e.g. convergence and stability) of numerical schemes. For pricing swings, the viscosity solution approach has been used in [16] and [17].

In this paper, the electricity price model we use is of a regime-switching mean-reverting type with two states, where the mean-reverting characteristic is added to the model adopted in [6], and this modification is motivated by empirical researches in [1] and [18], which report that electricity spot price is equipped with the mean-reversion feature. As for the swing option contract, we assume that there exist: (i) the local volume constraint, that is, the holder can either exercise one volume of spot or none each time she uses one right, and (ii) the refraction time constraint, which requires a fixed time break between two successive exercises. Under those settings, we formulate the pricing of the swing option as an optimal multiple stopping problem. Basing on the work of Carmona and Touzi [12], we reduce this problem. After the reduction, continuity and boundedness of value functions is obtained and then we try to characterize the value functions as the unique viscosity solution of the associated HJB variational inequalities. First, existence results can be received. In addition, we prove the comparison principle fitting this problem which ensure the uniqueness of the solutions in viscosity sense.

The structure of this paper is as follows. Section 2 introduces the model for the underlying electricity price and derives the variational inequalities that the value functions formally satisfy. Section 3 treats the properties of the value functions. Section 4 characterizes the value functions as viscosity solutions of the associated variational inequalities. Section 5 gives the comparison principle and the uniqueness result.

## Formulation of the problem

### Mean-reverting regime-switching model and swing options

For a fixed maturity *T*, we work on a probability space $(\varOmega,\mathcal{F},\{\mathcal{F}(t)\}_{t\in[0,T]},\mathbb{Q})$ and a real $\{\mathcal{F}(t)\}_{t\in[0,T]}$-adapted Brownian motion $\{ B(t),0\leq t\leq T\}$ in risk-neutral measure $\mathbb{Q}$. Let $\{ \alpha(t)\}_{0\leq t\leq T}$ be an independent time-homogeneous Markov chain with values in a finite state space $\mathcal{M}:=\{1,2\}$, and the related generator matrix $\mathbf{P}=(q_{ij})_{2\times2}$, satisfying $q_{ij}\geq0$ and $\sum_{j=1}^{2}q_{ij}=0$ for each $i,j\in \mathcal{M}$ and $i\neq j$.

We model the electricity price by the stochastic process $\{S(t)\} _{t\in[0,T]}$, which is governed by the following:

1 $$ dS(t)= \beta \bigl(\alpha(t) \bigr) \bigl(\xi \bigl(\alpha(t) \bigr)-S(t) \bigr)\,dt+\sigma \bigl(\alpha (t) \bigr)\,dB^{\mathbb{Q}}(t);\qquad S(0)=S_{0},\qquad \alpha(0)=i; $$

where $S_{0}$ and *i* are initial data; $\beta(i)$, $\xi(i)$ and $\sigma (i)$ for each $i\in\mathcal{M}$ are constants representing the speed of adjustment, the long-term mean level, and the volatility. Moreover, we notice that the usual Lipschitz and linear growth conditions are satisfied to ensure the existence and uniqueness of the strong solution to the SDE(1).

On the settings of swings, we follow [12]. Consider a swing option with $n\in\mathbb{N}^{+}$ exercise rights during the time period $[0,T]$. The strike price is $K\in\mathbb{R}^{+}$, thus the holder gets the payoff $\phi(x)=(K-x)^{+}$. All of the above parameters are fixed. And there are two rules the holder must obey:

1. Each time the holder use the right, she can exercise either one volume or none.
2. If one right is used at time *t*, the holder cannot use another right until after the time $t+\delta$, where $\delta>0$ is the refraction period.

We call the first item above the local volume constraint and the second the refraction time constraint.

### Variational inequalities of value functions

In this subsection, we start from introducing the value function of this problem. For simplicity, we sometimes use $f_{i}(t,s)$ to stand for any function $f(t,s,i):[0,T]\times\mathbb{R}^{+}\times \mathcal{M}\mapsto\mathbb{R}$.

#### Definition 1

Let $(t,s,i)\in[0, T]\times\mathbb{R}^{+}\times\mathcal{M}$. For fixed integer $n\in\mathbb{N}$, we define the value function

2 $$ V_{i}^{(n)}(t,s):=\sup_{\vec{\tau}\in\mathcal{T}_{t,T}^{(n)}} \mathbb {E} \Biggl[\sum_{k=1}^{n}e^{-r(\tau_{k}-t)} \phi \bigl(S(\tau_{k}) \bigr)\Big\vert S_{t} = s, \alpha_{t}=i \Biggr], $$

where $\mathcal{T}_{t,T}^{(n)}$ is the collection of $\vec{\tau}:=(\tau _{1},\tau_{2},\ldots,\tau_{n})$ having the following properties:

- $t\leq\tau_{1}\leq T$ a.s.,
- $\tau_{i}-\tau_{i-1}\geq\delta$ on $\{0\leq\tau_{i-1}\leq T\}$ a.s., $\forall i=1,2,\ldots,n$,
- $\tau_{i}:={T+}$ on $\{\tau_{i}>T\}$ a.s., $\forall i=1,2,\ldots,n$;

moreover, ${T+}>T$ is a cemetery time and we set $\phi(S_{T+})\equiv0$.

Here, the notation $V^{(n)}_{i}(t,s)$ denotes the value of swing option at time *t* with state value $(S(t),\alpha(t))=(s,i)$, and the superscript *n* is used to emphasize that the holder still has *n* rights before the expiry.

For the above value functions, we state in the following proposition an iterative equation showing the relationship between $V^{(n)}$ and $V^{(n-1)}$. It is this relationship that transforms the optimal multiple stopping problem into a sequence of optimal single stopping problems.

#### Proposition 1

*Let* $(t,s,i)\in[0, T]\times\mathbb{R}^{+}\times\mathcal{M}$. *For fixed integer* $n\in\mathbb{N}$, *the value function* $V_{i}^{(n)}$ *of* (2) *can be equivalently written follows*.

(i) *For* $n=0$,

3 $$ V_{i}^{(0)}(t,s) = 0. $$

(ii) *For* $n\geq1$,

4 $$ V_{i}^{(n)}(t,s)= \sup_{\tau\in\mathcal{T}_{t, T}}E^{\mathbb{Q}} \bigl[e^{-r(\tau-t)}G_{\alpha(\tau)}^{(n)} \bigl(\tau,S(\tau) \bigr) \vert S(t)=s,\alpha (t)=i \bigr], $$

*where* $\mathcal{T}_{t, T}$ *is the same as in the above definition*, *and*

5 $$ G_{i}^{(n)}(t,s):= \textstyle\begin{cases} (K-s)^{+}+E^{\mathbb{Q}} [e^{-r\delta}V_{\alpha(t+\delta )}^{(n-1)}(t+\delta,S(t+\delta))\vert S(t)=s,\alpha(t)=i ],\\ \quad t\in [0,T-\delta],\\ (K-s)^{+},\\ \quad t\in(T-\delta, T], \end{cases} $$

*where* *δ* *is the refraction time*.

#### Proof

Notice that the reward (or payoff) process $\phi (S_{t})=(K-S_{t})^{+}$ has continuous paths and the strong Markov property, which indicates that it satisfies conditions $(2.1)$, $(2.2)$ and $(2.8)$ in [12]. In addition, according to [19] we find that the filtration $\{\mathcal{F}(t)\} _{t\in[0,T]}$ satisfies the usual conditions and additional requirements $(2.10)$ in [12]. In this way, we can apply Theorem 1 in [12] to rewrite the problem (2) into the iteration form (4) with (5). □

#### Remark 1

The specific form of $G_{i}^{(n)}$ of (5) in Proposition 1 can be further written as follows.

(i) For $n=1$,

$$ G_{i}^{(1)}(t,s)=(K-s)^{+},\quad \forall t\in[0,T]. $$

(ii) For $n\geq2$, $G_{i}^{(n)}$ involves a European option price, i.e.,

$$ G_{i}^{(n)}(t,s):= \textstyle\begin{cases} (K-s)^{+}+f_{i}^{(n)}(0,s),&t\in[0,T-\delta],\\ (K-s)^{+},&t\in(T-\delta, T], \end{cases} $$

where $(f_{1}^{(n)}(\tau,s),f_{2}^{(n)}(\tau,s))$ satisfies

6 $$ \textstyle\begin{cases} \mathcal{L}_{1}f_{1}^{(n)}+q_{12}(f_{2}^{(n)}-f_{1}^{(n)})=0,\quad\tau\in[0,\delta ),s>0,\\ \mathcal{L}_{2}f_{2}^{(n)}+q_{21}(f_{1}^{(n)}-f_{2}^{(n)})=0,\quad\tau\in[0,\delta ),s>0,\\ f_{1}^{(n)}(\delta,\cdot)=f_{2}^{(n)}(\delta,\cdot)= \sum_{j=1}^{2}w_{ij}(\delta)V_{j}^{(n-1)}(t+\delta,\cdot),\quad s>0, \end{cases} $$

in which

$$ \mathcal{L}_{i}V_{i}^{(n)}:= \frac{\partial}{\partial t}V_{i}^{(n)}+\frac {1}{2} \sigma^{2}(i)\frac{\partial^{2}}{\partial s^{2}}V_{i}^{(n)}+\beta(i) \bigl(\xi (i)-s \bigr)\frac{\partial}{\partial s}V_{i}^{(n)}-rV_{i}^{(n)},\quad i\in\mathcal{M}. $$

To see how (6) comes about, we define

$$\begin{aligned} u_{i}^{(n)}(t,s):={}&E^{\mathbb{Q}} \Biggl[e^{-r\delta}\sum_{j=1}^{2}w_{ij}( \delta)V_{j}^{(n-1)} \bigl(t+\delta,S(t+\delta) \bigr)\Big\vert S(t)=s, \alpha(t)=i \Biggr], \\ & t\in[0, T-\delta], s\in\mathbb{R}^{+}, i=1,2. \end{aligned}$$

Let $f_{i}^{(n)}(0,s)=u_{i}^{(n)}(t,s)$, then using the Feynman–Kac formula, one gets what one wants.

Having transformed the problem in Proposition 1, now we show the PDE characterization of the value functions $V_{i}^{(n)}$ in the form of variational inequalities.

#### Theorem 1

*For* $n\in N^{+}$, *the pair* $(V_{1}^{(n)}(t,s), V_{2}^{(n)}(t,s))$ *formally satisfy the coupled variational inequalities*

7 $$ \textstyle\begin{cases} \min\{-\mathcal{L}_{1}V_{1}^{(n)}-q_{12}(V_{2}^{(n)}-V_{1}^{(n)}), V_{1}^{(n)}-G_{1}^{(n)}\}=0,\quad s>0, 0< t< T,\\ \min\{-\mathcal{L}_{2}V_{2}^{(n)}-q_{21}(V_{1}^{(n)}-V_{2}^{(n)}), V_{2}^{(n)}-G_{2}^{(n)}\}=0,\quad s>0, 0< t< T,\\ V_{1}^{(n)}=V_{2}^{(n)}=(K-s)^{+},\quad s>0,t=T, \end{cases} $$

*where* $\mathcal{L}_{i}V_{i}^{(n)}$ *and* $G_{i}^{(n)}$ *are the same as in Proposition *1.

#### Proof

Let $\zeta:[0,T]\times\mathbb{R}^{+}\times\mathcal{M}\mapsto\mathbb{R}$ be smooth function. Denote $\zeta_{i}(t,s):=\zeta(t,s,i)$. Let *τ* be a stopping time in $\mathcal{T}_{t,T}$. Then we have from the Dynkin formula that

$$\begin{aligned} &e^{-r(\tau-t)}\zeta \bigl(\tau,S(\tau), \alpha(\tau) \bigr)-\zeta \bigl(t,S(t),\alpha(t) \bigr) \\ &\quad= \int_{t}^{\tau}\,d \bigl(e^{-r(\theta-t)}\zeta \bigl( \theta, S(\theta),\alpha (\theta) \bigr) \bigr) \\ &\quad= \int_{t}^{\tau} \bigl[e^{-r(\theta-t)} \bigl( \mathcal{L}_{\alpha(\theta)}\zeta _{\alpha(\theta)}+q_{i1}( \zeta_{1}-\zeta_{2}) \bigr)\,d\theta+e^{-r(\theta -t)}\,dM( \theta) \bigr], \end{aligned}$$

where *M* is a martingale under measure $\mathbb{Q}$, and

$$\begin{aligned} \mathcal{L}_{i} \zeta_{i}:= &\frac{\partial}{\partial t}\zeta_{i}+\frac {1}{2} \sigma^{2}(i)\frac{\partial^{2}}{\partial s^{2}}\zeta_{i}+\beta(i) \bigl(\xi (i)-s \bigr)\frac{\partial}{\partial s}\zeta_{i}-r\zeta_{i},\quad i\in \mathcal{M}. \end{aligned}$$

Taking the expectation on both sides, we obtain

$$\begin{aligned} &\zeta_{i}(t,s) \\ &\quad =E^{\mathbb{Q}} \biggl[e^{-r(\tau-t)}\zeta_{\alpha(\tau)} \bigl(\tau, S( \tau ) \bigr)\\ &\qquad{}- \int_{t}^{\tau}e^{-r(\theta-t)} \bigl( \mathcal{L}_{\alpha(\theta)}\zeta _{\alpha(\theta)} + q_{ij} \bigl( \zeta_{j} \bigl(\theta,S(\theta) \bigr)-\zeta_{i} \bigl( \theta ,S(\theta) \bigr) \bigr) \bigr) \,d\theta\Big\vert S(t)=s,\alpha(t)=i \biggr], \end{aligned}$$

where $i,j\in\mathcal{M}, j\neq i$. Then under the assumption that

$$\begin{aligned} \zeta_{i}(\cdot,\cdot) \geq G_{i}^{(n)}(\cdot,\cdot) \quad\text{and}\quad {-}\mathcal{L}_{i} \zeta_{i}(\cdot,\cdot) - q_{ij} \bigl(\zeta_{j}( \cdot,\cdot) -\zeta_{i}(\cdot,\cdot) \bigr) \geq0,\quad i\in\mathcal{M}, \end{aligned}$$

we have

$$ \zeta_{i}(t,s)\geq E^{\mathbb{Q}} \bigl[e^{-r(\tau-t)}G_{\alpha(\tau )}^{(n)} \bigl(\tau,S(\tau) \bigr) \vert S(t)=s,\alpha(t)=i \bigr],\quad i\in\mathcal{M}, $$

and by taking the supreme on both sides, it follows that

$$ \zeta_{i}(t,s)\geq V_{i}^{(n)}(t,s). $$

Hence, we assume that $(\zeta_{1}(t,s),\zeta_{2}(t,s))$ is the solution of

$$ \textstyle\begin{cases} \min\{-\mathcal{L}_{i}\zeta_{i}-q_{ij}(\zeta_{j}-\zeta_{i}), \zeta _{i}-G_{i}^{(n)}\}=0,\quad 0< t< T, s>0, i,j\in\mathcal{M},j\neq i,\\ \zeta_{i}=G_{i}^{(n)}(s,i,T)=(K-s)^{+},\quad t=T, s>0, i\in\mathcal{M}. \end{cases} $$

On the other hand, define

$$ \tau^{*}:=\inf \bigl\{ t\leq\tau\leq T\vert\zeta_{\alpha(\tau)}^{(n)}( \tau ,S_{\tau})=G^{(n)} \bigl(\tau,S(\tau),\alpha(\tau) \bigr) \bigr\} \in\mathcal{T}_{t, T}, $$

which implies that

$$\begin{aligned} \zeta_{i}(t,s)={}&E^{\mathbb{Q}} \biggl[e^{-r(\tau^{*}-t)}\zeta_{\alpha(\tau ^{*})} \bigl(\tau^{*},S \bigl(\tau^{*} \bigr) \bigr)\\ &{}- \int_{t}^{\tau^{*}}e^{-r(\theta-t)}(\mathcal {L}_{\alpha(\theta)}\zeta_{\alpha(\theta)} +q_{ij}(\zeta_{j}- \zeta_{i}) \,d\theta\vert S(t)=s,\alpha(t)=i \biggr] \\ = {}&E^{\mathbb{Q}} \bigl[e^{-r(\tau^{*}-t)}G_{\alpha(\tau^{*})}^{(n)} \bigl( \tau ^{*},S \bigl(\tau^{*} \bigr) \bigr)\vert S(t)=s,\alpha(t)=i \bigr]\leq V_{i}^{(n)}(t,s). \end{aligned}$$

Above all, if $V^{(n)}$ is as smooth as *ζ*, then it is the solution of the problem (7). □

### Viscosity solutions

In this subsection, after introducing the notion of viscosity solution, we state that the value functions in this paper is the unique viscosity solution of the problem (7).

#### Definition 2

(Viscosity supersolution)

For $(t,s) \in[0,T]\times\mathbb{R}^{+}$, $i \in\mathcal{M}$ and $n \in N^{+}$, assume that $f_{i}^{(n)}(t,s)$ satisfies the following four conditions:

1. $f_{i}^{(n)}(t,s)$ is locally bounded.
2. $f_{i}^{(n)}(\cdot,\cdot)$ is continuous in $[0,T]\times\mathbb{R}^{+}$.
3. $f_{1}^{(n)}(T,s)=f_{2}^{(n)}(T,s)=(K-s)^{+}$ on $\{T\}\times\mathbb{R}^{+}$.
4. For $\forall(\bar{t},\bar{s})\in[0,T)\times\mathbb{R}^{+}$ and $\forall\phi\in C^{1,2}([0,T)\times\mathbb{R}^{+})$ such that
  $$ 0=f_{i}^{(n)}(\bar{t},\bar{s})-\phi(\bar{t},\bar{s})= \mathrm{strict}\min_{[0,T)\times\mathbb{R}^{+}} \bigl(f_{i}^{(n)}(t,s)- \phi(t,s) \bigr), $$
  we always have
  $$\begin{aligned} &\min \biggl\{ -\frac{\partial\phi(\bar{t},\bar{s})}{\partial t}-\frac {1}{2} \sigma^{2}(i)\frac{\partial^{2} \phi(\bar{t},\bar{s})}{\partial s^{2}}-\beta(i) \bigl(\xi(i)-\bar{s} \bigr) \frac{\partial\phi(\bar{t},\bar {s})}{\partial s}\\ &\quad {}+r\phi(\bar{t},\bar{s},i)-q_{ij} \bigl(f_{j}^{(n)}( \bar {t},\bar{s})-\phi(\bar{t},\bar{s}) \bigr), \phi(\bar{t},\bar{s})-G_{i}^{(n)}(\bar{t},\bar{s}) \biggr\} \geq0,\\ &\quad i,j\in \mathcal{M}, j\neq i, \end{aligned}$$
  where
  8 $$ G_{i}^{(n)}(\bar{t},\bar{s}):= \textstyle\begin{cases} (K-\bar{s})^{+}+E^{\mathbb{Q}}[e^{-r\delta}f_{\alpha(\bar{t}+\delta )}^{(n-1)}(\bar{t}+\delta,S(\bar{t}+\delta))\vert S(\bar{t})=\bar {s},\alpha(\bar{t})=i],\\ \quad \bar{t}\in[0,T-\delta],\\ (K-\bar{s})^{+},\\ \quad \bar{t}\in(T-\delta, T). \end{cases} $$

Then we say $f_{i}^{(n)}(t,s)$ is a continuous viscosity supersolution of the variational inequalities in (7) on $[0,T)\times\mathbb{R}^{+}$.

#### Definition 3

(Viscosity subsolution)

For $(t,s) \in[0,T]\times\mathbb{R}^{+}$, $i \in\mathcal{M}$ and $n \in N^{+}$, assume that $f_{i}^{(n)}(t,s)$ satisfies the following four conditions:

1. $f_{i}^{(n)}(t,s)$ is locally bounded.
2. $f_{i}^{(n)}(\cdot,\cdot)$ is continuous in $[0,T]\times\mathbb{R}^{+}$.
3. $f_{1}^{(n)}(T,s)=f_{2}^{(n)}(T,s)=(K-s)^{+}$ on $\{T\}\times\mathbb{R}^{+}$.
4. For $\forall(\bar{t},\bar{s})\in[0,T)\times\mathbb{R}^{+}$ and $\forall\phi\in C^{1,2}([0,T)\times\mathbb{R}^{+})$ such that
  $$ 0=f_{i}^{(n)}(\bar{t},\bar{s})-\phi(\bar{t},\bar{s})= \mathrm{strict}\max_{[0,T)\times\mathbb{R}^{+}} \bigl(f_{i}^{(n)}(t,s)- \phi(t,s) \bigr), $$
  we always have
  $$\begin{aligned} &\min \biggl\{ -\frac{\partial\phi(\bar{t},\bar{s})}{\partial t}-\frac {1}{2} \sigma^{2}(i)\frac{\partial^{2} \phi(\bar{t},\bar{s})}{\partial s^{2}}-\beta(i) \bigl(\xi(i)-\bar{s} \bigr) \frac{\partial\phi(\bar{t},\bar {s})}{\partial s}\\ &\quad +r\phi(\bar{t},\bar{s},i)-q_{ij} \bigl(f_{j}^{(n)}( \bar {t},\bar{s})-\phi(\bar{t},\bar{s}) \bigr), \phi(\bar{t},\bar{s})-G_{i}^{(n)}(\bar{t},\bar{s}) \biggr\} \leq0,\\ &\quad i,j\in \mathcal{M}, j\neq i, \end{aligned}$$
  where $G_{i}^{(n)}(\bar{t},\bar{s})$ is the same as in (8).

Then we say $f_{i}^{(n)}(t,s)$ is a continuous viscosity subsolution of the variational inequalities in (7) on $[0,T)\times\mathbb{R}^{+}$.

#### Definition 4

(Viscosity solution)

For $(t,s) \in[0,T]\times\mathbb{R}^{+}$, $i \in\mathcal{M}$ and $n \in N^{+}$, we say that $f_{i}^{(n)}(t,s)$ is a continuous viscosity solution of (7) on $[0,T]\times\mathbb{R}^{+}$ if it is both a continuous supersolution and a continuous subsolution of the variational inequalities in (7).

#### Theorem 2

*For* $(t,s) \in[0,T]\times\mathbb{R}^{+}$, $i \in\mathcal{M}$ *and* $n \in N^{+}$, $V_{i}^{(n)}(t,s)$ *defined in Definition *1 *is the unique viscosity solution of the problem* (7).

In Sects. 4 and 5, we will show the proofs of the existence and uniqueness results, respectively.

## Properties of the value function

In this part, we derive properties including continuity and local boundedness of the value function to demonstrate that it satisfies the first two conditions in Definition 2 (or in Definition 3, notice that they are the same).

### Lemma 1

*Let* $(t,s) \in[0,T]\times\mathbb{R}^{+}$, *then*, *for each* $i\in\mathcal {M}$ *and* $n\in N^{+}$, *the value function* $V_{i}^{(n)}(t,s)$ *is continuous with respect to* *s*.

### Proof

For each $i\in\mathcal{M}$, given $s_{1}$, $s_{2}$ and $t\in[0,T]$, let $S_{1}$ and $S_{2}$ be two solutions of the SDE (1) with $S_{1}(t)=s_{1}$ and $S_{2}(t)=s_{2}$, respectively. We adopt mathematical induction.

(i) For $n=1$, we have

9 $$\begin{aligned} &V^{(1)}(t,s_{1},i)- V^{(1)}(t,s_{2},i) \\ &\quad =\sup_{\tau\in\mathcal {T}_{t,T}}E^{\mathbb{Q}} \bigl[e^{-r(\tau-t)} \bigl(K-S_{1}(\tau) \bigr)^{+} \bigr]-\sup_{\tau\in \mathcal{T}_{t,T}}E^{\mathbb{Q}} \bigl[e^{-r(\tau-t)} \bigl(K-S_{2}(\tau) \bigr)^{+} \bigr] \\ &\quad \leq\sup_{\tau\in\mathcal{T}_{t,T}}E^{\mathbb{Q}} \bigl[e^{-r(\tau -t)} \bigl\vert \bigl(K-S_{1}(\tau) \bigr)^{+}- \bigl(K-S_{2}(\tau) \bigr)^{+} \bigr\vert \bigr] \\ &\quad \leq\sup_{\tau\in\mathcal{T}_{t,T}}E^{\mathbb{Q}} \bigl[e^{-r(\tau -t)} \bigl\vert S_{1}(\tau)-S_{2}(\tau) \bigr\vert \bigr]. \end{aligned}$$

Here we need to obtain the upper bound of $E^{\mathbb {Q}}[|S_{1}(u)-S_{2}(u)|]\ (u\in[t,T])$. From SDE (1), by using the Cauchy–Schwarz inequality and Tonelli’s theorem we find that

10 $$\begin{aligned} &E^{\mathbb{Q}} \bigl[ \bigl\vert S_{1}(u)-S_{2}(u) \bigr\vert ^{2} \bigr] \\ &\quad = E^{\mathbb{Q}} \biggl[ \biggl\vert (s_{1}-s_{2})+ \int_{t}^{u}\beta \bigl(\alpha(\tau) \bigr) \bigl(S_{2}(\tau)-S_{1}(\tau) \bigr)\,d\tau \biggr\vert ^{2} \biggr] \\ &\quad =E^{\mathbb{Q}} \biggl[(s_{1}-s_{2})^{2}+ 2(s_{1}-s_{2}) \int_{t}^{u}\beta \bigl(\alpha (\tau) \bigr) \bigl(S_{2}(\tau)-S_{1}(\tau) \bigr)\,d\tau \\ &\qquad{}+ \biggl( \int_{t}^{u}\beta \bigl(\alpha(\tau) \bigr) \bigl(S_{2}(\tau)-S_{1}(\tau) \bigr)\,d\tau \biggr)^{2} \biggr] \\ &\quad \leq 2 \vert s_{1}-s_{2} \vert ^{2}+2E^{\mathbb{Q}} \biggl[ \biggl( \int_{t}^{u}\beta \bigl(\alpha (\tau) \bigr) \bigl(S_{2}(\tau)-S_{1}(\tau) \bigr)\,d\tau \biggr)^{2} \biggr] \\ &\quad \leq 2 \vert s_{1}-s_{2} \vert ^{2}+CE^{\mathbb{Q}} \biggl[ \int_{t}^{u}\beta^{2} \bigl(\alpha(\tau ) \bigr) \bigl(S_{2}(\tau)-S_{1}(\tau) \bigr)^{2}\,d \tau \biggr] \\ &\quad \leq 2 \vert s_{1}-s_{2} \vert ^{2}+CE^{\mathbb{Q}} \biggl[ \int_{t}^{u} \bigl(S_{2}( \tau)-S_{1}(\tau ) \bigr)^{2}\,d\tau \biggr] \\ &\quad =2 \vert s_{1}-s_{2} \vert ^{2}+C \int_{t}^{u}E^{\mathbb{Q}} \bigl[ \bigl(S_{2}(\tau)-S_{1}(\tau ) \bigr)^{2} \bigr]\,d \tau. \end{aligned}$$

Notice that *C* is a generic positive constant, whose value varies in different cases. Next we adopt Gronwall’s inequality to deal with (10), and we obtain

$$\begin{aligned} E^{\mathbb{Q}} \bigl[ \bigl\vert S_{1}(u)-S_{2}(u) \bigr\vert ^{2} \bigr]\leq C \vert s_{1}-s_{2} \vert ^{2}, \quad \forall u\in[t,T]. \end{aligned}$$

Then, by using the Cauchy–Schwarz inequality again, we get an upper bound of $E^{\mathbb{Q}}[|S_{1}(u)-S_{2}(u)|]$, that is,

11 $$ E^{\mathbb{Q}} \bigl[ \bigl\vert S_{1}(u)-S_{2}(u) \bigr\vert \bigr]\leq C \vert s_{1}-s_{2} \vert ,\quad \forall t\in[0,T]. $$

With the inequality (11), we can continue to deal with the inequality (9) and finally get

$$ V^{(1)}(t,s_{1},i)- V^{(1)}(t,s_{2},i) \leq C \vert s_{1}-s_{2} \vert . $$

Hence $V^{(1)}(t,s,i)$ is (Lipschitz) continuous respect to *s*.

(ii) For $n=k$, $k\geq2$, we assume that

$$ V^{(k-1)} \bigl(t,S_{1}(t),i \bigr)- V^{(k-1)} \bigl(t,S_{2}(t),i \bigr)\leq C \vert s_{1}-s_{2} \vert ,\quad \forall t\in[0,T], $$

under which we can compute for $n=k$,

$$ \bigl\vert V^{(k)}(t,s_{1},i)- V^{(k)}(t,s_{2},i) \bigr\vert \leq\sup_{\tau\in\mathcal {T}_{t,T}}CE^{\mathbb{Q}} \bigl[ \bigl\vert G^{(k)} \bigl(\tau,S_{1}(\tau),\alpha(\tau ) \bigr)- G^{(k)} \bigl(\tau,S_{2}(\tau),\alpha(\tau) \bigr) \bigr\vert \bigr]. $$

Here we need to investigate the property of $|G^{(k)}(\tau,S_{1}(\tau ),\alpha(\tau))- G^{(k)}(\tau,S_{2}(\tau),\alpha(\tau))|$ as follows:

$$\begin{aligned} & \bigl\vert G^{(k)} \bigl( \tau,S_{1}( \tau),\alpha(\tau) \bigr)- G^{(k)} \bigl( \tau,S_{2}(\tau),\alpha (\tau) \bigr) \bigr\vert \\ &\quad \leq \bigl\vert \bigl(K-S_{1}(\tau) \bigr)^{+} - \bigl(K-S_{2}(\tau) \bigr)^{+} \bigr\vert \\ &\qquad{}+ \bigl\vert E^{\mathbb{Q}} \bigl[e^{-r\delta} \bigl(V^{(k-1)} \bigl(\tau+\delta ,S_{1}(\tau+\delta),\alpha(\tau+\delta) \bigr)-V^{(k-1)} \bigl(\tau+\delta,S_{2}(\tau +\delta),\alpha( \tau+\delta) \bigr) \bigr) \bigr] \bigr\vert \\ &\quad \leq C \bigl\vert S_{1}(\tau)-S_{2}(\tau) \bigr\vert +CE^{\mathbb{Q}} \bigl[ \bigl\vert V^{(k-1)} \bigl(\tau + \delta,S_{1}(\tau+\delta),\alpha(\tau+\delta) \bigr)\\ &\qquad{}-V^{(k-1)} \bigl(\tau+\delta ,S_{2}(\tau+\delta),\alpha(\tau+\delta) \bigr) \bigr\vert \bigr] \\ &\quad \leq C \bigl\vert S_{1}(\tau)-S_{2}(\tau) \bigr\vert +CE^{\mathbb{Q}} \bigl[ \bigl\vert S_{1}(\tau+\delta )-S_{2}(\tau+\delta) \bigr\vert \bigr] \\ &\quad \leq C \bigl\vert S_{1}(\tau)-S_{2}(\tau) \bigr\vert +C \vert s_{1}-s_{2} \vert . \end{aligned}$$

In this way, we get

$$ \bigl\vert V^{(k)}(t,s_{1},i)- V^{(k)}(t,s_{2},i) \bigr\vert \leq\sup_{\tau\in\mathcal {T}_{t,T}}E^{\mathbb{Q}} \bigl[C \bigl\vert S_{1}(\tau)-S_{2}(\tau) \bigr\vert +C \vert s_{1}-s_{2} \vert \bigr]\leq C \vert s_{1}-s_{2} \vert , $$

which completes the proof of continuity of $V^{(n)}(t,s,i)$ with respect to *s*. □

### Lemma 2

*Let* $(t,s)\in[0,T]\times\mathcal{\mathbb{R}^{+}}$, *then*, *for each* $i\in \mathcal{M}$ *and* $n\in N^{+}$, *the value function* $V_{i}^{(n)}(t,s)$ *is continuous with respect to* *t*.

### Proof

Firstly, we define the objective function

$$\begin{aligned} J^{(n)}(t,s,i,\tau)&:= E^{\mathbb{Q}} \bigl[e^{-r(\tau-t)}G^{(n)} \bigl(\tau,S(\tau ),\alpha(\tau) \bigr)\vert S(t)=s,\alpha(t)=i \bigr] \\ & =e^{rt}E^{\mathbb{Q}} \bigl[h^{(n)} \bigl(\tau, S(\tau) , \alpha(\tau) \bigr)\vert S(t)=s,\alpha(t)=i \bigr],\quad \tau\in[t,T], \end{aligned}$$

where

$$ h^{(n)} \bigl(\tau, S(\tau),\alpha(\tau) \bigr):=e^{-r\tau}G^{(n)} \bigl(\tau,S(\tau ),\alpha(\tau) \bigr). $$

Next, for any *t* and $t'$, we set $0< t< t'<\tau<\tau':=\tau+t'-t<T$, our discussion is based on mathematical induction.

(i) For $n=1$, we have

12 $$\begin{aligned} J^{(1)}(t,s,i,\tau) & :=e^{rt}E^{\mathbb{Q}} \bigl[h^{(1)} \bigl(\tau, S(\tau ), \alpha(\tau) \bigr)\vert S(t)=s,\alpha(t)=i \bigr] \\ & = e^{rt'}E^{\mathbb{Q}} \bigl[h^{(1)} \bigl( \tau', S \bigl(\tau' \bigr),\alpha \bigl( \tau' \bigr) \bigr)\vert S \bigl(t' \bigr)=s,\alpha \bigl(t' \bigr)=i \bigr] \\ & = e^{rt'}E^{\mathbb{Q}} \bigl[h^{(1)} \bigl(\tau+ \bigl(t'-t \bigr), S \bigl(\tau' \bigr), \alpha \bigl( \tau ' \bigr) \bigr)\vert S \bigl(t' \bigr)=s,\alpha \bigl(t' \bigr)=i \bigr], \end{aligned}$$

and

13 $$\begin{aligned} J^{(1)} \bigl(t',s,i, \tau' \bigr) & := e^{rt'}E^{\mathbb{Q}} \bigl[h^{(1)} \bigl(\tau', S \bigl(\tau ' \bigr),\alpha \bigl( \tau' \bigr) \bigr)\vert S \bigl(t' \bigr)=s,\alpha \bigl(t' \bigr)=i \bigr] \\ & =e^{rt}E^{\mathbb{Q}} \bigl[h^{(1)} \bigl(\tau, S(\tau), \alpha(\tau) \bigr)\vert S(t)=s,\alpha(t)=i \bigr] \\ & = e^{rt}E^{\mathbb{Q}} \bigl[h^{(1)} \bigl( \tau'- \bigl(t'-t \bigr), S(\tau), \alpha(\tau ) \bigr) \vert S(t)=s,\alpha(t)=i \bigr]. \end{aligned}$$

Then we investigate the property of $h^{(1)}(t,S(t),\alpha(t))\ (t\in [0,T])$. To this end, we assume that, for any $t_{1},t_{2}>0$, $S(t_{1})=s_{1}$, $S(t_{2})=s_{2}$, and since $|e^{-x_{1}}-e^{-x_{2}}|\leq |x_{1}-x_{2}|$ for any $x_{1},x_{2}\geq0$, we obtain

$$\begin{aligned} &h^{(1)} \bigl(t_{1},s_{1}, \alpha(t_{1}) \bigr)-h^{(1)} \bigl(t_{2},s_{2}, \alpha(t_{2}) \bigr)\\ &\quad \leq \bigl\vert h^{(1)} \bigl(t_{1},s_{1},\alpha(t_{1}) \bigr)-h^{(1)} \bigl(t_{2},s_{2}, \alpha(t_{2}) \bigr) \bigr\vert \\ &\quad = \bigl\vert e^{-rt_{1}} \bigl(K-S(t_{1}) \bigr)^{+} - e^{-rt_{2}} \bigl(K-S(t_{2}) \bigr)^{+} \bigr\vert \\ & \quad \leq \bigl\vert e^{-rt_{1}} \bigl(K-S(t_{1}) \bigr) - e^{-rt_{2}} \bigl(K-S(t_{2}) \bigr) \bigr\vert \\ &\quad = \bigl\vert K \bigl(e^{-rt_{1}}-e^{-rt_{2}} \bigr)+e^{-rt_{2}}S(t_{2})-e^{-rt_{1}}S(t_{1}) \bigr\vert \\ & \quad \leq K \bigl\vert e^{-rt_{1}}-e^{-rt_{2}} \bigr\vert + \bigl\vert e^{-rt_{2}}s_{2}-e^{-rt_{2}}s_{1}+e^{-rt_{2}}s_{1}-e^{-rt_{1}}s_{1} \bigr\vert \\ & \quad \leq K \bigl\vert e^{-rt_{1}}-e^{-rt_{2}} \bigr\vert +e^{-rt_{2}} \vert s_{2}-s_{1} \vert +s_{1} \bigl\vert e^{-rt_{2}}-e^{-rt_{1}} \bigr\vert \\ & \quad \leq K \vert t_{2}-t_{1} \vert +e^{-rt_{2}} \vert s_{2}-s_{1} \vert +s_{1} \vert t_{2}-t_{1} \vert \\ &\quad = C \vert t_{2}-t_{1} \vert +C \vert s_{2}-s_{1} \vert . \end{aligned}$$

Now we proceed to dealing with the equality (12) to find

14 $$\begin{aligned} &J^{(1)}(t,s,i,\tau) \\ &\quad \leq e^{rt'}E^{\mathbb{Q}} \bigl[h^{(1)} \bigl(\tau, S(\tau ), \alpha( \tau) \bigr)+C \bigl( \bigl\vert t'-t \bigr\vert \bigr)+C \bigl\vert S \bigl(\tau' \bigr)-S(\tau) \bigr\vert \vert S \bigl(t' \bigr)=s,\alpha \bigl(t' \bigr)=i \bigr]. \end{aligned}$$

With the fact that

$$ E^{\mathbb{Q}} \bigl[ \bigl\vert S \bigl(\tau' \bigr)-S(\tau) \bigr\vert \bigr]\leq\sqrt{E^{\mathbb{Q}} \bigl[ \bigl\vert S \bigl(\tau ' \bigr)-S(\tau) \bigr\vert ^{2} \bigr]}\leq C \bigl\vert \tau'-\tau \bigr\vert ^{\frac{1}{2}}=C \bigl\vert t'-t \bigr\vert ^{\frac{1}{2}}, $$

it follows from (14) that

$$\begin{aligned} &J^{(1)}(t,s,i,\tau) \\ &\quad\leq e^{rt'}E^{\mathbb{Q}} \bigl[h^{(1)} \bigl(\tau, S(\tau ),\alpha(\tau) \bigr)\vert S \bigl(t' \bigr)=s,\alpha \bigl(t' \bigr)=i \bigr]+C \bigl\vert t'-t \bigr\vert +C \bigl( \bigl\vert t'-t \bigr\vert ^{\frac {1}{2}} \bigr) \\ &\quad=J^{(1)} \bigl(t',s,i,\tilde{\tau}\bigr)+C \bigl\vert t'-t \bigr\vert +C \bigl\vert t'-t \bigr\vert ^{\frac{1}{2}} \\ &\quad=J^{(1)} \bigl(t',s,i,\tilde{\tau}\bigr)+C \bigl\vert t'-t \bigr\vert ^{\frac{1}{2}},\quad \tau\in[t,T],\tilde{\tau}\in \bigl[t',T \bigr]. \end{aligned}$$

Similarly, we can restart from the inequality (13) and get

$$\begin{aligned} J^{(1)} \bigl(t',s,i, \tau' \bigr) &\leq e^{rt}E^{\mathbb{Q}} \bigl[h \bigl( \tau, S(\tau),\alpha (\tau) \bigr)\vert S(t)=s,\alpha(t)=i \bigr]+C \bigl\vert t'-t \bigr\vert +C \bigl( \bigl\vert t'-t \bigr\vert ^{\frac{1}{2}} \bigr) \\ &=J^{(1)}(t,s,i,\tilde{\tau})+C \bigl\vert t'-t \bigr\vert +C \bigl\vert t'-t \bigr\vert ^{\frac{1}{2}} \\ &=J^{(1)}(t,s,i,\tilde{\tau})+C \bigl\vert t'-t \bigr\vert ^{\frac{1}{2}},\quad \tau'\in \bigl[t',T \bigr], \tilde{\tau}\in[t,T]. \end{aligned}$$

Finally, we get

$$\begin{aligned} & \bigl\vert V^{(1)} \bigl(t',s,i \bigr)-V^{(1)}(t,s,i) \bigr\vert \\ & \quad\leq\sup_{\hat{\tau}\in\mathcal {T}_{t',T}, \tau\in\mathcal{T}_{t,T}} \bigl\vert J^{(1)} \bigl(t',s,i,\hat{\tau}\bigr)-J^{(1)}(t,s,i,\tau) \bigr\vert \\ &\quad \leq C \bigl\vert t'-t \bigr\vert ^{\frac{1}{2}},\quad 0< t< t'< T, \end{aligned}$$

and this indicates the continuity of $V^{(1)}$ with respect to *t*.

(ii) For $n=k$, $k\geq2$, we assume that

$$ \bigl\vert V^{(k-1)} \bigl(t',s,i \bigr)-V^{(k-1)}(t,s,i) \bigr\vert \leq C \bigl\vert t'-t \bigr\vert ^{\frac{1}{2}},\quad 0< t< t'< T. $$

Then we start from defining

15 $$\begin{aligned} J^{(k)}(t,s,i,\tau) & :=e^{rt}E^{\mathbb{Q}} \bigl[h^{(k)} \bigl(\tau, S(\tau ), \alpha(\tau) \bigr)\vert S(t)=s,\alpha(t)=i \bigr] \\ & = e^{rt'}E^{\mathbb{Q}} \bigl[h^{(k)} \bigl( \tau', S \bigl(\tau' \bigr),\alpha \bigl( \tau' \bigr) \bigr)\vert S \bigl(t' \bigr)=s,\alpha \bigl(t' \bigr)=i \bigr] \\ & = e^{rt'}E^{\mathbb{Q}} \bigl[h^{(k)} \bigl(\tau+ \bigl(t'-t \bigr), S \bigl(\tau' \bigr),\alpha \bigl( \tau ' \bigr) \bigr)\vert S \bigl(t' \bigr)=s,\alpha \bigl(t' \bigr)=i \bigr] \end{aligned}$$

and

16 $$\begin{aligned} J^{(k)} \bigl(t',s,i, \tau' \bigr) & := e^{rt'}E^{\mathbb{Q}} \bigl[h^{(k)} \bigl(\tau', S \bigl(\tau ' \bigr),\alpha \bigl( \tau' \bigr) \bigr)\vert S \bigl(t' \bigr)=s,\alpha \bigl(t' \bigr)=i \bigr] \\ & =e^{rt}E^{\mathbb{Q}} \bigl[h^{(k)} \bigl(\tau, S(\tau), \alpha(\tau) \bigr)\vert S(t)=s,\alpha(t)=i \bigr] \\ & = e^{rt}E^{\mathbb{Q}} \bigl[h^{(k)} \bigl( \tau'- \bigl(t'-t \bigr), S(\tau), \alpha(\tau ) \bigr) \vert S(t)=s,\alpha(t)=i \bigr]. \end{aligned}$$

Next we need to investigate the property of $h^{(k)}(t,S(t),\alpha (t))\ (t\in[0,T])$. For this purpose, let $t_{1},t_{2}>0$, $S(t_{1})=s_{1}$, $S(t_{2})=s_{2}$, and we estimate

$$\begin{aligned} &h^{(k)} \bigl(t_{1},s_{1}, \alpha(t_{1}) \bigr)-h^{(k)} \bigl(t_{2},s_{2}, \alpha(t_{2}) \bigr)\\ &\quad \leq \bigl\vert h^{(k)} \bigl(t_{1},s_{1},\alpha(t_{1}) \bigr)-h^{(k)} \bigl(t_{2},s_{2}, \alpha(t_{2}) \bigr) \bigr\vert \\ & \quad =\bigl\vert e^{-rt_{1}}G^{(k)} \bigl(t_{1},S(t_{1}), \alpha (t_{1}) \bigr)-e^{-rt_{2}}G^{(k)} \bigl(t_{2},S(t_{2}),\alpha(t_{2}) \bigr) \bigr\vert \\ &\quad \leq \bigl\vert e^{-rt_{1}}G^{(k)} \bigl(t_{1},S(t_{1}), \alpha (t_{1}) \bigr)-e^{-rt_{1}}G^{(k)} \bigl(t_{2},S(t_{2}),\alpha(t_{2}) \bigr) \bigr\vert \\ & \qquad{}+ \bigl\vert e^{-rt_{1}}G^{(k)} \bigl(t_{2},S(t_{2}), \alpha (t_{1}) \bigr)-e^{-rt_{2}}G^{(k)} \bigl(t_{2},S(t_{2}),\alpha(t_{2}) \bigr) \bigr\vert \\ &\quad:=I_{1}+I_{2}, \end{aligned}$$

here we need to deal with $I_{1}$ and $I_{2}$. Note that

$$\begin{aligned} I_{1}={}& \bigl\vert e^{-rt_{1}}G^{(k)} \bigl(t_{1},S(t_{1}),\alpha (t_{1}) \bigr)-e^{-rt_{1}}G^{(k)} \bigl(t_{2},S(t_{2}), \alpha(t_{2}) \bigr) \bigr\vert \\ \leq{}&e^{-rt_{1}} \bigl\vert G^{(k)} \bigl(t_{1},S(t_{1}), \alpha (t_{1}) \bigr)-G^{(k)} \bigl(t_{2},S(t_{2}), \alpha(t_{2}) \bigr) \bigr\vert \\ ={}&C \bigl\vert G^{(k)} \bigl(t_{1},S(t_{1}), \alpha(t_{1}) \bigr)-G^{(k)} \bigl(t_{2},S(t_{2}), \alpha(t_{2}) \bigr) \bigr\vert \\ \leq{}& C \bigl( \bigl\vert G^{(k)} \bigl(t_{1},S(t_{1}), \alpha(t_{1}) \bigr)-G^{(k)} \bigl(t_{1},S(t_{2}), \alpha (t_{2}) \bigr) \bigr\vert \\ &{}+ \bigl\vert G^{(k)} \bigl(t_{1},S(t_{2}), \alpha(t_{1}) \bigr)-G^{(k)} \bigl(t_{2},S(t_{2}), \alpha(t_{2}) \bigr) \bigr\vert \bigr) \\ :={}&C(I_{11}+I_{12}), \end{aligned}$$

where

$$\begin{aligned} I_{11}={}& \bigl\vert G^{(k)} \bigl(t_{1},S(t_{1}),\alpha(t_{1}) \bigr)-G^{(k)} \bigl(t_{1},S(t_{2}),\alpha (t_{2}) \bigr) \bigr\vert \\ ={}& \bigl\vert G^{(k)} \bigl(t_{1},s_{1}, \alpha(t_{1}) \bigr)-G^{(k)} \bigl(t_{1},s_{2}, \alpha (t_{1}) \bigr) \bigr\vert \\ ={}& \bigl\vert (K-s_{1})^{+}+E^{\mathbb{Q}} \bigl[e^{-r\delta}V^{(k-1)} \bigl(t_{1}+\delta ,S_{1}(t_{1}+\delta), \alpha(t_{1}+\delta) \bigr) \bigr] \\ &{}-(K-s_{2})^{+}-E^{\mathbb{Q}} \bigl[e^{-r\delta}V^{(k-1)} \bigl(t_{1}+\delta ,S_{2}(t_{1}+\delta),\alpha(t_{1}+\delta) \bigr) \bigr] \bigr\vert \\ \leq{}& \vert s_{2}-s_{1} \vert +CE^{\mathbb{Q}} \bigl[ \bigl\vert V^{(k-1)} \bigl(t_{1}+\delta,S_{1}(t_{1}+ \delta ),\alpha(t_{1}+\delta) \bigr) \\ &{}-V^{(k-1)} \bigl(t_{1}+\delta,S_{2}(t_{1}+\delta), \alpha(t_{1}+\delta) \bigr) \bigr\vert \bigr] \\ \leq{}& \vert s_{2}-s_{1} \vert +CE^{\mathbb{Q}} \bigl[ \bigl\vert S_{2}(t_{1}+\delta)-S_{1}(t_{1}+ \delta) \bigr\vert \bigr] \\ \leq{}&C \vert s_{2}-s_{1} \vert \end{aligned}$$

and

$$\begin{aligned} I_{12}={}& \bigl\vert G^{(k)} \bigl(t_{1},S(t_{2}),\alpha(t_{1}) \bigr)-G^{(k)} \bigl(t_{2},S(t_{2}),\alpha (t_{2}) \bigr) \bigr\vert \\ ={}& \bigl\vert E^{\mathbb{Q}} \bigl[e^{-r\delta}V^{(k-1)} \bigl(t_{1}+\delta,S(t_{1}+\delta),\alpha (t_{1}+ \delta) \bigr)|S(t_{1})=s_{2},\alpha(t_{1})=i \bigr] \\ &{}-E^{\mathbb{Q}} \bigl[e^{-r\delta}V^{(k-1)} \bigl(t_{2}+ \delta,S(t_{2}+\delta),\alpha (t_{2}+ \delta) \bigr)|S(t_{2})=s_{2},\alpha(t_{2})=i \bigr] \bigr\vert \\ ={}& \bigl\vert E^{\mathbb{Q}} \bigl[e^{-r\delta}V^{(k-1)} \bigl(t_{1}+\delta ,S^{t_{1},s_{2},i}(t_{1}+\delta), \alpha(t_{1}+\delta) \bigr) \bigr] \\ &{}-E^{\mathbb{Q}} \bigl[e^{-r\delta}V^{(k-1)} \bigl(t_{2}+\delta ,S^{t_{2},s_{2},i}(t_{2}+\delta), \alpha(t_{2}+\delta) \bigr) \bigr] \bigr\vert \\ \leq{}&E^{\mathbb{Q}} \bigl[C \bigl\vert V^{(k-1)} \bigl(t_{1}+ \delta,S^{t_{1},s_{2},i}(t_{1}+\delta ),\alpha(t_{1}+\delta) \bigr)\\ &{}-V^{(k-1)} \bigl(t_{2}+\delta,S^{t_{2},s_{2},i}(t_{2}+ \delta ),\alpha(t_{2}+\delta) \bigr) \bigr\vert \bigr] \\ \leq{}&E^{\mathbb{Q}} \bigl[C \vert t_{2}-t_{1} \vert ^{\frac{1}{2}} \bigr] \quad\bigl(\text{since } S^{t_{1},s_{2},i}(t_{1}+ \delta)=S^{t_{2},s_{2},i}(t_{2}+\delta) \bigr) \\ \leq{}&C \vert t_{2}-t_{1} \vert ^{\frac{1}{2}}, \end{aligned}$$

and in this way we get

$$\begin{aligned} I_{1}&= \bigl\vert e^{-rt_{1}}G^{(k)} \bigl(t_{1},S(t_{1}),\alpha (t_{1}) \bigr)-e^{-rt_{1}}G^{(k)} \bigl(t_{2},S(t_{2}), \alpha(t_{2}) \bigr) \bigr\vert \\ &\leq C \vert s_{2}-s_{1} \vert +C \vert t_{2}-t_{1} \vert ^{\frac{1}{2}}. \end{aligned}$$

For $I_{2}$, we get

$$\begin{aligned} I_{2}&= \bigl\vert e^{-rt_{1}}G^{(k)} \bigl(t_{2},S(t_{2}),\alpha (t_{2}) \bigr)-e^{-rt_{2}}G^{(k)} \bigl(t_{2},S(t_{2}), \alpha(t_{2}) \bigr) \bigr\vert \\ &\leq G^{(k)} \bigl(t_{2},S(t_{2}), \alpha(t_{2}) \bigr) \bigl\vert e^{-rt_{1}}-e^{-rt_{2}} \bigr\vert \\ &\leq C \bigl\vert e^{-rt_{1}}-e^{-rt_{2}} \bigr\vert \\ &\leq C \vert t_{2}-t_{1} \vert . \end{aligned}$$

Hence we can conclude that

$$\begin{aligned} \bigl\vert h^{(k)} \bigl(t_{1},s_{1}, \alpha(t_{1}) \bigr)-h^{(k)} \bigl(t_{2},s_{2}, \alpha(t_{2}) \bigr) \bigr\vert \leq C \vert s_{2}-s_{1} \vert +C \vert t_{2}-t_{1} \vert ^{\frac{1}{2}}+C \vert t_{2}-t_{1} \vert . \end{aligned}$$

Now we come back to Eq. (15), and we estimate

$$\begin{aligned} &J^{(k)}(t,s,i,\tau) \\ &\quad\leq e^{rt'}E^{\mathbb{Q}} \bigl[h^{(k)} \bigl(\tau, S(\tau ) \bigr)+C \bigl\vert S \bigl( \tau' \bigr)-S(\tau) \bigr\vert +C \bigl\vert t'-t \bigr\vert ^{\frac{1}{2}}+C \bigl\vert t'-t \bigr\vert \vert S \bigl(t' \bigr)=s,\alpha \bigl(t' \bigr)=i \bigr]. \end{aligned}$$

With the fact that

$$\begin{aligned} E^{\mathbb{Q}} \bigl[ \bigl\vert S \bigl(\tau' \bigr)-S(\tau) \bigr\vert \bigr]\leq\sqrt{E^{\mathbb{Q}} \bigl[ \bigl\vert S \bigl(\tau ' \bigr)-S(\tau) \bigr\vert ^{2} \bigr]}\leq C \bigl\vert \tau'-\tau \bigr\vert ^{\frac{1}{2}}=C \bigl\vert t'-t \bigr\vert ^{\frac{1}{2}}, \end{aligned}$$

we find that

$$\begin{aligned} J^{(k)}(t,s,i,\tau) &\leq e^{rt'}E^{\mathbb{Q}} \bigl[h^{(k)} \bigl(\tau, S(\tau ),\alpha(\tau) \bigr)\vert S \bigl(t' \bigr)=s,\alpha \bigl(t' \bigr)=i \bigr]+C \bigl\vert t'-t \bigr\vert +C \bigl( \bigl\vert t'-t \bigr\vert ^{\frac {1}{2}} \bigr) \\ &=J^{(k)} \bigl(t',s,i,\tilde{\tau}\bigr)+C \bigl\vert t'-t \bigr\vert +C \bigl\vert t'-t \bigr\vert ^{\frac{1}{2}} \\ &=J^{(k)} \bigl(t',s,i,\tilde{\tau}\bigr)+C \bigl\vert t'-t \bigr\vert ^{\frac{1}{2}},\quad \tau\in[t,T],\tilde{\tau}\in \bigl[t',T \bigr]. \end{aligned}$$

Similarly, we can restart from the equality (16) to get

$$\begin{aligned} J^{(k)} \bigl(t',s,i, \tau' \bigr) &\leq e^{rt}E^{\mathbb{Q}} \bigl[h \bigl( \tau, S(\tau),\alpha (\tau) \bigr)\vert S(t)=s,\alpha(t)=i \bigr]+C \bigl\vert t'-t \bigr\vert +C \bigl( \bigl\vert t'-t \bigr\vert ^{\frac{1}{2}} \bigr) \\ &=J^{(k)}(t,s,i,\tilde{\tau})+C \bigl\vert t'-t \bigr\vert +C \bigl\vert t'-t \bigr\vert ^{\frac{1}{2}} \\ &=J^{(k)}(t,s,i,\tilde{\tau})+C \bigl\vert t'-t \bigr\vert ^{\frac{1}{2}},\quad \tau'\in \bigl[t',T \bigr], \tilde{\tau}\in[t,T]. \end{aligned}$$

In this way, we finally arrive at the estimation that

$$\begin{aligned} \bigl\vert V^{(k)} \bigl(t',s,i \bigr)-V^{(k)}(t,s,i) \bigr\vert & \leq\sup_{\hat{\tau}\in\mathcal {T}_{t',T}, \tau\in\mathcal{T}_{t,T}} \bigl\vert J^{(k)} \bigl(t',s,i,\hat{\tau}\bigr)-J^{(k)}(t,s,i,\tau) \bigr\vert \\ & \leq C \bigl\vert t'-t \bigr\vert ^{\frac{1}{2}}, \end{aligned}$$

and this indicates the continuity of $V^{(k)}$ with respect to *t*. Therefore we complete the proof of the continuity of $V^{(n)}$ with respect to *t*. □

### Lemma 3

*Let* $(t,s) \in[0,T]\times\mathbb{R}^{+}$, *then*, *for each* $i\in\mathcal {M}$ *and* $n\in N^{+}$, *there exists a constant* *C* *such that* $|V^{(n)}(t,s,i)|\leq C(1+|s|)$.

### Proof

For each $i\in\mathcal{M}$, given any $t\in[0,T]$, assume that $S(t)=s$ and $\alpha(t)=i$. Then, by mathematical induction, we have the following.

(i) For $n=1$,

$$\begin{aligned} \bigl\vert V^{(1)}(t,s,i) \bigr\vert &=\sup _{\tau\in\mathcal{T}_{t, T}}E^{\mathbb {Q}} \bigl[e^{-r(\tau-t)}G_{j}^{(1)} \bigl(\tau,S(\tau) \bigr)\vert S(t)=s,\alpha(t)=i \bigr] \\ &=\sup_{\tau\in\mathcal{T}_{t, T}}E^{\mathbb{Q}} \bigl[e^{-r(\tau -t)} \bigl(K-S( \tau) \bigr)^{+}\vert S(t)=s,\alpha(t)=i \bigr] \\ &\leq\sup_{\tau\in\mathcal{T}_{t, T}}E^{\mathbb{Q}} \bigl[C \bigl\vert K-S(\tau) \bigr\vert \vert S(t)=s,\alpha(t)=i \bigr] \\ &\leq\sup_{\tau\in\mathcal{T}_{t, T}}E^{\mathbb {Q}} \bigl[C \bigl( \bigl\vert K-S(t) \bigr\vert + \bigl\vert S(t)-S(\tau) \bigr\vert \bigr)\vert S(t)=s, \alpha(t)=i \bigr] \\ &\leq\sup_{\tau\in\mathcal{T}_{t, T}}E^{\mathbb {Q}} \bigl[C \bigl(K+ \vert s \vert + \bigl\vert S(t)-S(\tau) \bigr\vert \bigr)\vert S(t)=s,\alpha(t)=i \bigr] \\ &=\sup_{\tau\in\mathcal{T}_{t, T}} C \bigl(K+ \vert s \vert \bigr)+E^{\mathbb {Q}} \bigl[C \bigl\vert S(t)-S(\tau) \bigr\vert \vert S(t)=s,\alpha(t)=i \bigr] \\ &\leq\sup_{\tau\in\mathcal{T}_{t, T}}C \bigl(K+ \vert s \vert \bigr)+ C \vert \tau-t \vert ^{\frac {1}{2}}\leq C \bigl(K+ \vert s \vert \bigr)+ CT^{\frac{1}{2}}=C \bigl(1+ \vert s \vert \bigr). \end{aligned}$$

(ii) For $n=k$, $k\geq2$, assuming that

$$\begin{aligned} \bigl\vert V^{(k-1)}(t,s,i) \bigr\vert \leq C \bigl(1+ \vert s \vert \bigr), \quad \forall t\in[0,T], \end{aligned}$$

then using the results in Lemma 1 and Lemma 2, we get

$$\begin{aligned} \bigl\vert V^{(k)}(t,s,i) \bigr\vert ={}& \sup _{\tau\in\mathcal{T}_{t, T}}E^{\mathbb {Q}} \bigl[e^{-r(\tau-t)}G^{(k)} \bigl(\tau,S(\tau),\alpha(\tau) \bigr)\vert S(t)=s,\alpha (t)=i \bigr] \\ \leq{}& \sup_{\tau\in\mathcal{T}_{t, T}} \bigl(CE^{\mathbb{Q}} \bigl[ \bigl(K-S(\tau ) \bigr)^{+}\vert S(t)=s,\alpha(t)=i \bigr] \\ &{}+CE^{\mathbb{Q}} \bigl[ \bigl\vert V^{(k-1)} \bigl(\tau+\delta,S( \tau+ \delta ),j \bigr) \bigr\vert |S(t)=s,\alpha(t)=i \bigr] \bigr) \\ \leq{}& C \bigl(1+ \vert s \vert \bigr)+\sup_{\tau\in\mathcal{T}_{t, T}}E^{\mathbb {Q}} \bigl[C \bigl( \bigl\vert V^{(k-1)}(t,s,j) \bigr\vert +C \vert \tau+ \delta-t \vert ^{\frac{1}{2}} \\ &{}+C \bigl\vert S(\tau+\delta)-S(t) \bigr\vert \bigr)|S(t)=s,\alpha(t)=i \bigr] \\ \leq{}&C \bigl(1+ \vert s \vert \bigr)+\sup_{\tau\in\mathcal{T}_{t, T}}E^{\mathbb {Q}}[C \bigl(C \bigl(1+ \vert s \vert \bigr)+ \vert \tau+\delta-t \vert ^{\frac{1}{2}}+C \vert \tau+\delta-t \vert ^{\frac {1}{2}} \bigr) \\ \leq{}&C \bigl(1+ \vert s \vert \bigr). \end{aligned}$$

 □

## Existence of the viscosity solution

This section shows the existence result. We break the whole proof into two parts, where the first part corresponds to the supersolution property of the value function, and in the second part we deal with the subsolution property.

### Lemma 4

*For any* $n\in\mathbb{N}^{+}$, $(V^{(n)}(t,s,1),V^{(n)}(t,s,2))$ *is the viscosity supersolution of* (7).

### Proof

We only need to show that, for any $i\in\mathcal{M}$, $V^{(n)}(t,s,i)$ is a viscosity supersolution to

17 $$ \textstyle\begin{cases} \min\{-\mathcal{L}_{i}W-q_{ij}(V_{j}^{(n)}-W), W-G_{i}^{(n)}\}=0, \quad s>0, 0< t< T,\\ W=(K-s)^{+},\quad s>0,t=T, \end{cases} $$

where $\mathcal{L}_{i}V$ and $G_{i}^{(n)}$ are the same as in Propersition 1.

Given $(\bar{t},\bar{s})\in[0,T)\times\mathbb{R}^{+}$, assume that $\alpha(\bar{t})=i\in\mathcal{M}$, and let $\phi\in C^{1,2}([0,T)\times \mathbb{R}^{+})$ such that

18 $$ 0=V^{(n)}(\bar{t},\bar{s},i)-\phi(\bar{t},\bar{s})= \mathrm{strict}\min_{(t,s)\in(0,T]\times\mathbb{R}^{+}} \bigl(V^{(n)}(t,s,i)-\phi(t,s) \bigr). $$

Let $\{(t_{m},s_{m})\}$ be a sequence in $[0,T]\times\mathbb{R}^{+}$ such that

$$ \bigl\{ (t_{m},s_{m}) \bigr\} \rightarrow(\bar{t},\bar{s}) \quad\text{and} \quad V_{i}^{(n)}(t_{m},s_{m}) \rightarrow V_{i}^{(n)}(\bar{t},\bar{s}). $$

Since $\phi\in C^{1,2}$, we have

19 $$ \eta_{m}:=V^{(n)}(t_{m},s_{m},i)- \phi(t_{m},s_{m})\rightarrow0. $$

Next, let *θ* be the time such that $\alpha(t)\equiv i$ in $[\bar {t},\theta]$. In addition, we denote by $S^{m}(t):=S^{t_{m},s_{m},i}(t)$ the associated state process with initial data $S^{m}(t_{m})=s_{m}$. For *m* sufficiently large, we have

$$ 0< h_{m}:=\eta_{m}^{\frac{1}{2}}\mathbf{1}_{\{\eta_{m}\neq0\}} + \frac {1}{m}\mathbf{1}_{\{\eta_{m}=0\}}\leq\theta-t_{m}. $$

Then we define the stopping time

$$ \gamma_{m}:=\inf \bigl\{ t>t_{m}: \bigl(t-t_{m},S^{m}(t)-s_{m} \bigr)\notin[0,h_{m})\times[-\alpha ,\alpha] \bigr\} , $$

where $\alpha>0$ is some given constant. By the dynamic programming principle we have

$$ V^{(n)}(t_{m},s_{m},i)\geq E^{\mathbb{Q}} \bigl[e^{-r(\gamma_{m}-t_{m})}V \bigl(\gamma _{m},S^{m}( \gamma_{m}),i \bigr) \bigr], $$

and, together with (18) and (19), we have

20 $$ 0\leq E^{\mathbb{Q}} \bigl[\eta_{m}+ \phi(t_{m},s_{m})-e^{-r(\gamma_{m}-t_{m})}\phi \bigl( \gamma_{m},S^{m}(\gamma_{m}) \bigr) \bigr]. $$

Similar to [20], we set

$$ \psi \bigl(t,S(t),\alpha(t) \bigr)= \textstyle\begin{cases} \phi(t,S(t)), & \mbox{if } \alpha(t)=i, \\ V^{(n)}(t,S(t),\alpha(t)), & \mbox{otherwise}. \end{cases} $$

Therefore by Dynkin’s formula, we continue the inequality (20) as follows:

$$\begin{aligned} 0 \leq{}& \eta_{m}+E^{\mathbb{Q}} \bigl[ \psi(t_{m},s_{m},i)-e^{-r(\gamma_{m}-t_{m})}\psi \bigl( \gamma_{m},S^{m}(\gamma_{m}),i \bigr) \bigr] \\ ={}& \eta_{m}-E^{\mathbb{Q}} \biggl[ \int_{t_{m}}^{\gamma_{m}}\,d \bigl(e^{-r(\tau-t_{m})}\psi \bigl( \tau,S^{m}(\tau),i \bigr) \bigr) \biggr] \\ ={}&\eta_{m}-E^{\mathbb{Q}} \biggl[ \int_{t_{m}}^{\gamma_{m}}e^{-r(\tau-t_{m})} \biggl( \frac{\partial\psi(\tau,S^{m}(\tau),i)}{\partial t} +\frac{1}{2}\sigma ^{2}(i)\frac{\partial^{2}\psi(\tau,S^{m}(\tau),i)}{\partial s^{2}}\\ &{}+ \beta(i) \bigl(\xi (i)-S^{m}(\tau) \bigr)\frac{\partial\psi(\tau,S^{m}(\tau),i)}{\partial s} \\ &{}-r\psi \bigl(\tau,S^{m}(\tau),i \bigr) + q_{ij} \bigl(\psi \bigl(\tau,S^{m}(\tau),j \bigr)-\psi \bigl(\tau ,S^{m}(\tau),i \bigr) \bigr) \biggr)\,dt \biggr]\\ &{}+E^{\mathbb{Q}} \biggl[ \int_{t_{m}}^{\gamma _{m}}e^{-r(\tau-t_{m})}\,dM(\tau) \biggr] \\ ={}&\eta_{m}-E^{\mathbb{Q}} \biggl[ \int_{t_{m}}^{\gamma_{m}}e^{-r(\tau-t_{m})} \biggl( \frac{\partial\psi(\tau,S^{m}(\tau),i)}{\partial t}+\frac{1}{2}\sigma ^{2}(i) \frac{\partial^{2}\psi(\tau,S^{m}(\tau),i)}{\partial s^{2}}\\ &{}+ \beta(i) \bigl(\xi (i)-S^{m}(\tau) \bigr)\frac{\partial\psi(\tau,S^{m}(\tau),i)}{\partial s} \\ &{}-r\psi \bigl(\tau,S^{m}(\tau),i \bigr) + q_{ij} \bigl(V^{(n)} \bigl(\tau,S^{m}(\tau),j \bigr)-\psi \bigl( \tau,S^{m}(\tau),i \bigr) \bigr) \biggr)\,dt \biggr],\quad j\neq i. \end{aligned}$$

Next we divide both sides by $\gamma_{m}-t_{m}$, then the mean value theorem leads us to

21 $$\begin{aligned} &\frac{\eta_{m}}{\gamma_{m}-t_{m}}-e^{-r(\tau-t_{m})} ( \frac{\partial\psi (\tau,S^{m}(\tau),i)}{\partial t}+\frac{1}{2}\sigma^{2}(i)\frac{\partial ^{2}\psi(\tau,S^{m}(\tau),i)}{\partial s^{2}} \\ &\quad {}+ \beta(i) \bigl(\xi(i)-S^{m}(\tau) \bigr)\frac {\partial\psi(\tau,S^{m}(\tau),i)}{\partial s} \\ &\quad{}-r\psi \bigl(\tau,S^{m}(\tau),i \bigr) \\ &\quad{}+ q_{ij} \bigl(V^{(n)} \bigl(\tau,S^{m}(\tau),j \bigr)-\psi \bigl( \tau,S^{m}(\tau),i \bigr) \bigr)\geq0, \quad\tau\in[t_{m}, \gamma_{m}], j\neq i, \end{aligned}$$

Note that, for *m* sufficiently large, $\gamma_{m}=t_{m}+h_{m}$, therefore

$$\begin{aligned} \frac{\eta_{m}}{\gamma_{m}-t_{m}}=\frac{\eta_{m}}{h_{m}}= \textstyle\begin{cases} 0\cdot\frac{1}{m}, & \mbox{if } \eta_{m}=0, \\ \sqrt{\eta_{m}}, & \mbox{otherwise}. \end{cases}\displaystyle \end{aligned}$$

Finally, we send *m* to infinity, and the inequality (21) becomes

$$\begin{aligned} &{-}\frac{\partial\phi(\bar{t},\bar{s})}{\partial t}-\frac{1}{2}\sigma ^{2}(i)\frac{\partial^{2}\phi(\bar{t},\bar{s})}{\partial s^{2}}-\beta(i) \bigl(\xi (i)-\bar{s} \bigr) \frac{\partial\phi(\bar{t},\bar{s})}{\partial s}+r\phi(\bar {t},\bar{s}) - q_{ij} \bigl(V^{(n)}(\bar{t},\bar{s},j)-\phi(\bar{t},\bar {s}) \bigr)\\ &\quad \geq0,\quad j\neq i. \end{aligned}$$

In addition, Theorem 1 states

$$\begin{aligned} V^{(n)}(\bar{t},\bar{s},i)-G^{(n)}(\bar{t},\bar{s},i)\geq0 \end{aligned}$$

and together with Lemma 1, we find that $V^{(n)}(t,s,i)$ is the continuous viscosity supersolution of (17). □

### Lemma 5

*For any* $n\in\mathbb{N}^{+}$, $(V^{(n)}(t,s,1),V^{(n)}(t,s,2))$ *is the viscosity subsolution of* (7).

### Proof

We only need to show that, for any $i\in\mathcal{M}$, $V^{(n)}(t,s,i)$ is a viscosity subsolution to

22 $$\begin{aligned} \textstyle\begin{cases} \min\{-\mathcal{L}_{i}W-q_{ij}(V_{j}^{(n)}-W), W-G_{i}^{(n)}\}=0, \quad s>0, 0< t< T,\\ W=(K-s)^{+},\quad s>0,t=T, \end{cases}\displaystyle \end{aligned}$$

where $\mathcal{L}_{i}V$ and $G_{i}^{(n)}$ are the same as in Lemma 1.

We argue by contradiction. Given $(\bar{t},\bar{s})\in[0,T)\times \mathbb{R}^{+}$, assume $\alpha(\bar{t})=i\in\mathcal{M}$ and let $\phi ^{1,2}([0,T)\times\mathbb{R}^{+})$ such that

23 $$\begin{aligned} 0=V^{(n)}(\bar{t},\bar{s},i)-\phi(\bar{t},\bar{s})= \mathrm{strict}\max_{(t,s)\in(0,T]\times\mathbb{R}^{+}} \bigl(V^{(n)}(t,s,i)-\phi(t,s) \bigr). \end{aligned}$$

We assume to the contrary that

$$\begin{aligned} &{-}\frac{\partial\phi(\bar{t},\bar{s})}{\partial t}-\frac{1}{2}\sigma ^{2}(i)\frac{\partial^{2}\phi(\bar{t},\bar{s})}{\partial s^{2}}-\beta(i) \bigl(\xi (i)-\bar{s} \bigr) \frac{\partial\phi(\bar{t},\bar{s})}{\partial s}+r\phi(\bar {t},\bar{s}) - q_{ij} \bigl(V^{(n)}(\bar{t},\bar{s},j)-\phi(\bar{t},\bar {s}) \bigr)\\ &\quad > 0,\quad j\neq i, \end{aligned}$$

and

$$\begin{aligned} V^{(n)}(\bar{t},\bar{s},i)-g(\bar{t},\bar{s},i)> 0. \end{aligned}$$

Next, let *θ* be the time such that $\alpha(t)\equiv i$ in $[\bar {t},\theta]$. We try to work towards a contradiction of the weak dynamic programming principle, i.e.,

24 $$\begin{aligned} & V^{(n)}(\bar{t},\bar{s},i) \\ &\quad \leq E^{\mathbb{Q}} \bigl[e^{-r(\tau\wedge\tau'-\bar {t})} \bigl(\mathbf{1}_{\{\tau< \tau'\}}G^{(n)} \bigl( \tau,S( \tau),i \bigr) \\ &\qquad{}+\mathbf {1}_{\{\tau\geq\tau'\}}V^{(n)} \bigl( \tau',S \bigl(\tau' \bigr),i \bigr) \bigr)|S(\bar{t})= \bar {s},\alpha( \bar{t})=i \bigr] \end{aligned}$$

for all $(\bar{t},\bar{s})\in[0,T]\times\mathbb{R}^{+}$ and $\tau'\in \mathcal{T}_{\bar{t},\theta}$ such that $S^{\bar{t},\bar{s}}(\tau')$ is $L^{\infty}$-bounded.

Firstly by the properties that $G^{(n)}\in C([0,T]\times\mathbb {R}^{+}\times\mathcal{M})$ and the first equality of (23), we know that there exist $\delta>0$ and $0< h\leq\theta$ such that the following inequalities hold:

25 $$\begin{aligned} &{-}\frac{\partial\phi(\bar{t},\bar{s})}{\partial t}-\frac{1}{2} \sigma ^{2}(i)\frac{\partial^{2}\phi(\bar{t},\bar{s})}{\partial s^{2}}-\beta(i) \bigl(\xi (i)-\bar{s} \bigr) \frac{\partial\phi(\bar{t},\bar{s})}{\partial s} \\ &\quad{}+r\phi(\bar {t},\bar{s}) - q_{ij} \bigl(V^{(n)}(\bar{t},\bar{s},j)-\phi(\bar{t},\bar {s}) \bigr)\geq0,\quad j\neq i \end{aligned}$$

and

26 $$\begin{aligned} \phi(t,s)-G^{(n)}(t,s,i)\geq V^{(n)}(t,s,i)-G^{(n)}(t,s,i) \geq\delta \end{aligned}$$

on $D :=[\bar{t},\bar{t}+h]\times[\bar{s}-\delta,\bar{s}+\delta]$. Secondly, by the second equality of (23), we get

27 $$\begin{aligned} -\gamma:=\max_{\partial D} \bigl(V^{(n)}( \cdot, \cdot,i)-\phi(\cdot,\cdot,i) \bigr)< 0. \end{aligned}$$

Thirdly, by the continuity of $V^{(n)}(t,s,i)$, there exists a sequence $\{(t_{m},s_{m})\}$ in $(0,T]\times\mathbb{R}^{+}$ such that

$$\begin{aligned} (t_{m},s_{m})\rightarrow(\bar{t},\bar{s}) \quad\text{and}\quad V(t_{m},s_{m},i)\rightarrow V(\bar{t},\bar{s},i)\quad \text{as } m \rightarrow \infty. \end{aligned}$$

Next we define the stopping time,

$$\begin{aligned} \gamma_{m}:=\inf \bigl\{ t>t_{m}| \bigl(t,S^{t_{m},s_{m}}(t) \bigr)\notin D \bigr\} , \end{aligned}$$

and denote

28 $$\begin{aligned} \eta_{m}:= V^{(n)}(t_{m}, s_{m}, i)-\phi(t_{m}, s_{m})\rightarrow0. \end{aligned}$$

Then, for arbitrary stopping time $\tau\in\mathcal{T}_{t_{m},T}$,

$$\begin{aligned} & V^{(n)}(t_{m}, s_{m}, i)= \eta_{m}+\phi(t_{m}, s_{m}). \end{aligned}$$

And we define

$$\begin{aligned} \psi \bigl(t,S(t),\alpha(t) \bigr)= \textstyle\begin{cases} \phi(t,S(t)), & \mbox{if } \alpha(t)=i, \\ V^{(n)}(t,S(t),\alpha(t)), & \mbox{otherwise}. \end{cases}\displaystyle \end{aligned}$$

Therefore,

$$\begin{aligned} &V^{(n)}(t_{m}, s_{m}, i)\\ &\quad= \eta_{m}+\phi(t_{m}, s_{m}) \\ &\quad =\eta_{m}+E^{\mathbb{Q}} \biggl[e^{-r(\tau\wedge\gamma_{m}-t_{m})}\psi \bigl(\tau \wedge\gamma_{m},S(\tau\wedge\gamma_{m}),i \bigr) - \int_{t_{m}}^{\tau\wedge\gamma_{m}}e^{-r(t-t_{m})} \biggl( \frac{\partial\psi (t,S(t),i)}{\partial t} \\ &\qquad{}+\frac{1}{2}\sigma^{2}(i)\frac{\partial^{2}\phi(t,S(t),i)}{\partial s^{2}}+\beta(i) \bigl( \xi(i)-S(t) \bigr)\frac{\partial\psi(t,S(t),i)}{\partial s}-r\psi \bigl(t,S(t),i \bigr) \\ &\qquad{} + q_{ij} \bigl(\psi \bigl(t, S(t),j \bigr)-\psi \bigl(t, S(t),i \bigr) \bigr) \biggr)\,dt\\ &\qquad{}+ \int _{t_{m}}^{\tau\wedge\gamma_{m}}e^{-r(t-t_{m})}\,dM(t)|S(t_{m})=s_{m}, \alpha (t_{m})=i \biggr],\quad j\in\mathcal{M},j\neq\alpha(t) \\ &\quad =\eta_{m}+E^{\mathbb{Q}} \biggl[e^{-r(\tau\wedge\gamma_{m}-t_{m})}\psi \bigl(\tau \wedge\gamma_{m},S(\tau\wedge\gamma_{m}),i \bigr) - \int_{t_{m}}^{\tau\wedge\gamma_{m}}e^{-r(t-t_{m})} \biggl( \frac{\partial\psi (t,S(t),i)}{\partial t} \\ &\qquad{}+\frac{1}{2}\sigma^{2}(i)\frac{\partial^{2}\phi(t,S(t),i)}{\partial s^{2}}+\beta(i) \bigl( \xi(i)-S(t) \bigr)\frac{\partial\psi(t,S(t),i)}{\partial s}-r\psi \bigl(t,S(t),i \bigr) \\ &\qquad{} + q_{ij} \bigl(V^{(n)} \bigl(t, S(t),j \bigr)-\psi \bigl(t, S(t),i \bigr) \bigr) \biggr)\,dt|S(t_{m})=s_{m}, \alpha(t_{m})=i \biggr],\quad j\in\mathcal{M},j\neq\alpha(t). \end{aligned}$$

Noting that the process $(t,S^{t_{m},s_{m},i}(t))$ is in *D*, then, by (25), (26) and (27) we find that

$$\begin{aligned} &V^{(n)}(t_{m}, s_{m}, i) \\ &\quad \geq\eta_{m}+E^{\mathbb{Q}} \bigl[e^{-r(\tau \wedge\gamma_{m}-t_{m})}\psi \bigl(\tau \wedge\gamma_{m},S(\tau\wedge\gamma_{m}),i \bigr) |S(t_{m})=s_{m},\alpha(t_{m})=i \bigr] \\ &\quad =\eta_{m}+E^{\mathbb{Q}} \bigl[e^{-r(\tau\wedge\gamma_{m}-t_{m})} \bigl(\psi \bigl( \tau,S(\tau),i \bigr)\mathbf {1}_{\{\tau< \gamma_{m}\}} +\psi \bigl(\gamma_{m},S( \gamma_{m}),i \bigr)\mathbf{1}_{\{\tau\geq\gamma_{m}\}} \bigr) |\\ &\qquad S(t_{m})=s_{m},\alpha(t_{m})=i \bigr] \\ &\quad =\eta_{m}+E^{\mathbb{Q}} \bigl[e^{-r(\tau\wedge\gamma_{m}-t_{m})} \bigl(\phi \bigl( \tau,S(\tau) \bigr)\mathbf {1}_{\{\tau< \gamma_{m}\}} +\phi \bigl(\gamma_{m},S( \gamma_{m}) \bigr)\mathbf{1}_{\{\tau\geq\gamma_{m}\}} \bigr) |\\ &\qquad S(t_{m})=s_{m}, \alpha(t_{m})=i \bigr] \\ &\quad \geq \eta_{m}+E^{\mathbb{Q}} \bigl[e^{-r(\tau\wedge\gamma_{m}-t_{m})} \bigl( \bigl(G^{(n)} \bigl(\tau,S(\tau ),i \bigr)+\delta \bigr) \mathbf{1}_{\{\tau< \gamma_{m}\}}\\ &\qquad{} + \bigl(V^{(n)} \bigl(\gamma_{m},S( \gamma_{m}),i \bigr)+\gamma \bigr)\mathbf{1}_{\{\tau\geq \gamma_{m}\}} \bigr)|S(t_{m})=s_{m},\alpha(t_{m})=i \bigr] \\ &\quad \geq\eta_{m}+ \delta\wedge\gamma+E^{\mathbb{Q}} \bigl[e^{-r(\tau\wedge\gamma_{m}-t_{m})} \bigl( \bigl(G^{(n)} \bigl(\tau,S(\tau ),i \bigr) \bigr)\mathbf{1}_{\{\tau< \gamma_{m}\}} \\ &\qquad{}+ \bigl(V^{(n)} \bigl( \gamma_{m},S(\gamma_{m}),i \bigr) \bigr)\mathbf{1}_{\{\tau\geq\gamma _{m}\}} \bigr)|S(t_{m})=s_{m},\alpha(t_{m})=i \bigr], \end{aligned}$$

since $\eta_{m}\rightarrow0$ as $m\rightarrow\infty$, and *τ* is arbitrary. This provides the contradiction of (24). □

## Uniqueness of the viscosity solution

The main task of this section is to prove the comparison principle of the HJB variational inequality problem (7), which ensures the uniqueness of the solution.

Before that, we try to prove the following comparison principle of the HJB equation problem:

$$\begin{aligned} (\mathbb{P}) \textstyle\begin{cases} &F_{1}(t,s,\rho_{1},q,p,M):=-q+(r+q_{12})\rho_{1}-\mathcal{H}_{1}(t,s,p,M)=0,\quad (t,s)\in[0,T)\times\mathbb{R}^{+} , \\ &F_{2}(t,s,\rho_{2},q,p,M):=-q+(r+q_{21})\rho_{2}-\mathcal{H}_{2}(t,s,p,M)=0,\quad (t,s)\in[0,T)\times\mathbb{R}^{+} , \\ &\rho_{1}(T,s)=\rho_{2}(T,s)=(K-s)^{+},\quad s\in\mathbb{R}^{+}, \end{cases}\displaystyle \end{aligned}$$

where

$$\begin{aligned} \mathcal{H}_{i}(t,s,p,M):=-q_{ij}\rho_{j}(t,s)+ \beta_{i}(\xi_{i}-s)p-\frac {\sigma^{2}}{2}M,\quad i\in\mathcal{M}. \end{aligned}$$

Since the problem $(\mathbb{P})$ is of the weakly coupled type, we only need to consider the problem

$$\begin{aligned} \bigl(\mathbb{P'} \bigr) \textstyle\begin{cases} F_{i}(t,s,W,q,p,M)\\ \quad:=-q+(r+q_{ij})W-\mathcal{H}_{i}(t,s,p,M)=0,\quad j\neq i,(t,s)\in[0,T)\times\mathbb{R}^{+} , \\ W(T,s)=(K-s)^{+},\quad s\in\mathbb{R}^{+}, \end{cases}\displaystyle \end{aligned}$$

where *V* is as above. To begin with, we introduce Definitions 5, 6,7 and Ishii’s lemma, which will play important roles in the succeeding proof. For more details as regards the above definitions and Ishii’s lemma, we refer to the classical literature [21] and [22].

### Definition 5

Let $U_{i}$ be upper semicontinous (u.s.c.), $\phi\in C^{1,2}([0,T]\times \mathbb{R}^{+})$, and $(\bar{t},\bar{s})\in[0,T)\times\mathbb{R}^{+}$ be a maximum point of $U_{i}-\phi$. Define a second-order superjet of $U_{i}$ at $(\bar{t},\bar{s})$ as

$$\begin{aligned} \mathcal{P}^{2,+}U_{i}(\bar{t},\bar{s}):={}& \biggl\{ (q,p,M)\in R\times R\times \mathcal{S}|U_{i}(t,s)\leq U_{i}(\bar{t}, \bar{s})+q(t-\bar{t})+p(s-\bar {s})\\ &{}+\frac{1}{2}M(s-\bar{s})^{2}+o \bigl( \vert t-\bar{t} \vert + \vert s-\bar{s} \vert ^{2} \bigr) \biggr\} . \end{aligned}$$

### Definition 6

Let $V_{i}$ be lower semicontinuous (l.s.c.), $\phi\in C^{1,2}([0,T]\times\mathbb{R}^{+})$, and $(\bar{t},\bar{s})\in [0,T)\times\mathbb{R}^{+}$ be a maximum point of $V_{i}-\phi$. Define a second-order subjet of $V_{i}$ at $(\bar{t},\bar{s})$ as

$$\begin{aligned} \mathcal{P}^{2,-}V_{i}(\bar{t},\bar{s}):={}& \biggl\{ (q,p,M)\in \mathbb{R}\times \mathbb{R}\times\mathcal{S}|V_{i}(t,s)\geq V_{i}(\bar{t},\bar{s})+q(t-\bar {t})+p(s-\bar{s})\\ &{}+ \frac{1}{2}M(s-\bar{s})^{2}+o \bigl( \vert t-\bar{t} \vert + \vert s-\bar {s} \vert ^{2} \bigr) \biggr\} . \end{aligned}$$

### Definition 7

An u.s.c. (resp. l.s.c.) function $w_{i}$ on $[0,T)\times\mathbb{R}^{+}$ is a viscosity subsolution (resp. supersolution) of $(\mathbb{P})$ on $[0,T)\times\mathbb{R}^{+}$ if and only if for all $(t,s)\in[0,T)\times \mathbb{R}^{+}$ and all $(q,p,M)\in\mathcal{P}^{2,+}w(t,s)$ (resp. $\mathcal {P}^{2,-}w_{i}(t,s)$),

$$\begin{aligned} F \bigl(t,s,w_{i}(t,s),q,p,M \bigr)\leq0 \quad (\mbox{resp. }\geq0). \end{aligned}$$

### Lemma 6

(Ishii’s lemma)

*Let* $U_{i}(V_{i})$ *be a u*.*s*.*c*. (*l*.*s*.*c*.) *function on* $[0,T)\times\mathbb {R}^{+}$, $\varphi\in C^{1,1,2,2}([0,T)^{2}\times\mathbb{R}^{+}\times\mathbb {R}^{+})$ *and* $(\bar{t},\bar{\tau},\bar{x},\bar{y})\in[0,T)^{2}\times \mathbb{R}^{+}\times\mathbb{R}^{+}$ *a local maximum* (*minimum*) *of* $U_{i}(t,x)-V_{i}(\tau,y)-\varphi(t,\tau,x,y)$. *Then*, *for all* $\eta>0$, *there exist* $M,N\in\mathcal{S}$ *satisfying*

$$\begin{aligned} & \biggl(\frac{\partial\varphi}{\partial t}(\bar{t},\bar{\tau},\bar {x}, \bar{y}), D_{x}\varphi(\bar{t},\bar{\tau},\bar{x},\bar{y}), M \biggr) \in \mathcal{P}^{2,+}U_{i}(\bar{t},\bar{x}) , \\ & \biggl(-\frac{\partial\varphi}{\partial\tau}(\bar{t},\bar{\tau},\bar {x},\bar{y}), -D_{y}\varphi(\bar{t},\bar{\tau},\bar{x},\bar{y}), N \biggr)\in \mathcal{P}^{2,-}V_{i}(\bar{\tau},\bar{y}), \end{aligned}$$

*and*

$$\left(\begin{array}{cc} M & 0\\ 0 & -N \end{array}\right)\leq D_{xy}^{2}\varphi (\bar{t},\bar{\tau },\bar{x},\bar{y})+\eta {\left(D_{xy}^{2}\varphi (\bar{t},\bar{\tau },\bar{x},\bar{y})\right)}^{2}.$$

Now we proceed to the statement of the comparison principle for the problem $(\mathbb{P'})$, and the proof of it.

### Theorem 3

*Let* $U_{i}$ *be a u*.*s*.*c*. *viscosity subsolution of problem* $(\mathbb{P'})$ *with polynomial growth condition*, *and let* $V_{i}$ *be l*.*s*.*c*. *viscosity supersolution of problem* $(\mathbb{P'})$ *with polynomial growth condition*. *In addition*, *let* $U_{i}(T,s)=V_{i}(T,s)=(K-s)^{+}$ *for* $s\in \mathbb{R}^{+}$. *If* $U_{i}(T,\cdot)\leq V_{i}(T,\cdot)$, *then* $U_{i}\leq V_{i}$ *in* $[0,T)\times\mathbb{R}^{+}$.

### Proof

We argue by contradiction.

Step 1. Assume that the supreme of $U_{i}-V_{i}$ on $[0,T]\times\mathbb {R}^{+}$ is attained at $(\bar{t},\bar{x})\in[0,T)\times\mathcal{O}$ for some bounded set $\mathcal{O}\subset\mathbb{R}^{+}$, i.e.,

29 $$\begin{aligned} M:=\sup_{[0,T]\times\mathbb{R}^{+}}(U_{i}-V_{i})= \max_{[0,T)\times\mathcal {O}}(U_{i}-V_{i})=(U_{i}-V_{i}) (\bar{t},\bar{x}), \end{aligned}$$

and suppose that

30 $$\begin{aligned} M>0. \end{aligned}$$

Then, for any $\epsilon>0$, we define

$$\begin{aligned} \varPhi_{\epsilon}(t,\tau,x,y)= U_{i}(t,x)-V_{i}( \tau,y)-\varphi_{\epsilon}(t_{\epsilon},\tau_{\epsilon},x_{\epsilon},y_{\epsilon}) \end{aligned}$$

with

31 $$\begin{aligned} \varphi_{\epsilon}(t,\tau,x,y)=\frac{1}{2\epsilon} \bigl[(t- \tau )^{2}+(x-y)^{2} \bigr]\geq0. \end{aligned}$$

Similar to (29), we denote

32 $$\begin{aligned} M_{\epsilon}:=\sup_{[0,T]^{2}\times\mathcal{O}^{2}} \varPhi_{\epsilon}=U_{i}(t_{\epsilon},x_{\epsilon})-V_{i}( \tau_{\epsilon},y_{\epsilon})-\varphi _{\epsilon}(t_{\epsilon}, \tau_{\epsilon},x_{\epsilon},y_{\epsilon}). \end{aligned}$$

In the sequel, we try to show

33 $$\begin{aligned} M_{\epsilon}\rightarrow M,\qquad \varphi_{\epsilon}\rightarrow0, \quad \mbox{as }\epsilon \rightarrow0. \end{aligned}$$

Note that

$$\begin{aligned} M\leq M_{\epsilon}, \end{aligned}$$

which implies that

$$\begin{aligned} (U_{i}-V_{i}) (\bar{t},\bar{x})\leq U_{i}(t_{\epsilon},x_{\epsilon})-V_{i}( \tau _{\epsilon},y_{\epsilon})-\varphi_{\epsilon}(t_{\epsilon}, \tau_{\epsilon},x_{\epsilon},y_{\epsilon}), \end{aligned}$$

i.e.,

34 $$\begin{aligned} \varphi_{\epsilon}(t_{\epsilon},\tau_{\epsilon},x_{\epsilon},y_{\epsilon}) \leq U_{i}(t_{\epsilon},x_{\epsilon})-V_{i}( \tau_{\epsilon},y_{\epsilon})-(U_{i}-V_{i}) \bigl(t^{*},s^{*} \bigr), \end{aligned}$$

which indicates that $\varphi_{\epsilon}(t_{\epsilon},\tau_{\epsilon},x_{\epsilon},y_{\epsilon})$ is bounded. Then sending $\epsilon\rightarrow 0^{+}$, we find from (31) and (34) that

35 $$\begin{aligned} (t_{\epsilon},\tau_{\epsilon},x_{\epsilon},y_{\epsilon}) \rightarrow(\bar {t},\bar{\tau},\bar{x},\bar{y})\in[0,T]^{2}\times \mathcal{O}^{2} \quad\text{and}\quad \bar{t}=\bar{\tau},\qquad \bar{x}=\bar{y}. \end{aligned}$$

Hence,

36 $$\begin{aligned} 0\leq\lim_{\epsilon\rightarrow0}\varphi_{\epsilon}(t_{\epsilon}, \tau _{\epsilon},x_{\epsilon},y_{\epsilon})\leq(U_{i}-V_{i}) (\bar{t},\bar {x})-(U-V) (\bar{t},\bar{x})\leq0, \end{aligned}$$

which implies $\varphi_{\epsilon}\rightarrow0$ as $\epsilon\rightarrow0$. From (32), (35), (36), $M_{\epsilon}\rightarrow M$.

In view of Ishii’s lemma, there exist $M,N\in\mathcal{S}$ satisfying

$$\left(\begin{array}{cc} M & 0\\ 0 & -N \end{array}\right)\leq \frac{3}{\epsilon }\left(\begin{array}{cc} 1 & -1\\ -1 & 1 \end{array}\right),$$

which implies that

$$\begin{aligned} \sigma^{2}(x_{\epsilon})M-\sigma^{2}(y_{\epsilon})N \leq\frac{3}{\epsilon} \bigl\vert \sigma ^{2}(x_{\epsilon})- \sigma^{2}(y_{\epsilon}) \bigr\vert ^{2}=0 \quad\bigl(\text{since } \sigma ^{2}(x_{\epsilon})=\sigma^{2}(y_{\epsilon}) \bigr), \end{aligned}$$

and

$$\begin{aligned} & \biggl(\frac{1}{\epsilon}(t_{\epsilon}- \tau_{\epsilon}),\frac{1}{\epsilon }(x_{\epsilon}-y_{\epsilon}),M \biggr)\in\mathcal{P}^{2,+}U(t_{\epsilon},x_{\epsilon}), \\ & \biggl(\frac{1}{\epsilon}(t_{\epsilon}-\tau_{\epsilon}), \frac{1}{\epsilon }(x_{\epsilon}-y_{\epsilon}),N \biggr)\in \mathcal{P}^{2,-}V(\tau_{\epsilon},y_{\epsilon}). \end{aligned}$$

Step 2. Since $U_{i},V_{i}$ are, respectively, a subsolution and a supersolution of $(\mathbb{P'})$, we have

$$\begin{aligned} & {-}\frac{1}{\epsilon}(t_{\epsilon}- \tau_{\epsilon})+(r+q_{ij}) U_{i}(t_{\epsilon},x_{\epsilon})- \mathcal{H}_{i} \biggl(t_{\epsilon},x_{\epsilon}, \frac {1}{\epsilon}(x_{\epsilon}-y_{\epsilon}),M \biggr)\leq0, \\ & {-}\frac{1}{\epsilon}(t_{\epsilon}-\tau_{\epsilon})+(r+q_{ij}) V_{i}(\tau _{\epsilon},y_{\epsilon})-\mathcal{H}_{i} \biggl(\tau_{\epsilon},y_{\epsilon},\frac {1}{\epsilon}(x_{\epsilon}-y_{\epsilon}),N \biggr)\geq0, \end{aligned}$$

which leads to

$$\begin{aligned} &(r+q_{ij}) \bigl(U_{i}(t_{\epsilon},x_{\epsilon})- V_{i}(\tau_{\epsilon},y_{\epsilon}) \bigr) \\ & \quad \leq \mathcal{H}_{i} \biggl(t_{\epsilon},x_{\epsilon}, \frac{1}{\epsilon}(x_{\epsilon}-y_{\epsilon}),M \biggr) - \mathcal{H}_{i} \biggl(\tau_{\epsilon},y_{\epsilon}, \frac {1}{\epsilon}(x_{\epsilon}-y_{\epsilon}),N \biggr) \\ &\quad \leq q_{ij} \bigl(V_{j}(\tau_{\epsilon},y_{\epsilon})-U_{j}(t_{\epsilon},x_{\epsilon}) \bigr)-\frac{1}{\epsilon}(x_{\epsilon}-y_{\epsilon})^{2}+ \frac{\sigma^{2}}{2}(M-N) \\ &\quad \leq q_{ij}C \bigl( \vert t_{\epsilon}-\tau_{\epsilon} \vert ^{\frac{1}{2}}+ \vert x_{\epsilon}-y_{\epsilon} \vert \bigr)-\frac{1}{\epsilon} \vert x_{\epsilon}-y_{\epsilon} \vert ^{2}. \end{aligned}$$

Finally, after sending *ϵ* to 0, we conclude that

$$\begin{aligned} (r+q_{ij})M=(r+q_{ij}) \bigl(U_{i}(\bar{t}, \bar{x})- V_{i}(\bar{\tau},\bar{y}) \bigr) \leq0, \end{aligned}$$

which contradicts the assumption (30). □

Now we consider the corresponding coupled variational inequalities problem,

$$\begin{aligned} (\mathbb{C}) \textstyle\begin{cases} \min\{F_{1}(t,s,\rho_{1},q,p,M), \rho_{1}-G_{1}^{(n)}\}=0,\quad (t,s)\in[0,T)\times \mathbb{R}^{+} , \\ \min\{F_{2}(t,s,\rho_{2},q,p,M), \rho_{2}-G_{2}^{(n)}\}=0, \quad (t,s)\in[0,T)\times \mathbb{R}^{+} , \\ \rho_{1}(T,s)=\rho_{2}(T,s)=(K-s)^{+},\quad s\in\mathbb{R}^{+}. \end{cases}\displaystyle \end{aligned}$$

Similarly, we only need to consider the problem

$$\begin{aligned} (\mathbb{UNC}) \textstyle\begin{cases} \min\{F_{i}(t,s,W,q,p,M), W-G_{i}^{(n)}\}=0, \quad j\neq i,(t,s)\in[0,T)\times \mathbb{R}^{+} , \\ W(T,s)=(K-s)^{+},\quad s\in\mathbb{R}^{+}. \end{cases}\displaystyle \end{aligned}$$

### Theorem 4

*Let* $U_{i}$ *be a u*.*s*.*c*. *viscosity subsolution of problem* $(\mathbb{UNC})$ *with polynomial growth condition*, *and let* $V_{i}$ *be l*.*s*.*c*. *viscosity supersolution of problem* $(\mathbb{UNC})$ *with polynomial growth condition*. *If* $U_{i}(T,\cdot)\leq V_{i}(T,\cdot)$, *then* $U_{i}\leq V_{i}$ *in* $[0,T)\times\mathbb{R}^{+}$.

### Proof

The only difference appears at Step 2 in the proof of Theorem 3, that is,

$$\begin{aligned} & \min \bigl\{ F_{i}(t_{\epsilon},x_{\epsilon},W,q,p,M), U_{i}-G_{i}^{(n)} \bigr\} \leq0 , \\ & \min \bigl\{ F_{i}(\tau_{\epsilon},y_{\epsilon},W,q,p,M), V_{i}-G_{i}^{(n)} \bigr\} \geq0. \end{aligned}$$

These two inequalities lead to the following two cases:

1. Either $U_{i}-G_{i}^{(n)}\leq0$. Together with the inequality $V_{i}-G_{i}^{(n)}\geq0$ leads to contradiction of (29).
2. Or $F_{i}(t_{\epsilon},x_{\epsilon},W,q,p,M)\leq0$, which can be combined with the supersolution part $F_{i}(\tau_{\epsilon},y_{\epsilon},W,q,p,M)\geq0$ exactly as in the proof of Theorem 3, and this leads to the same contradiction as above.

 □
